# Dietary Application of Synergistically Degraded Low-Molecular-Weight Chitosan to Promote Health and Antioxidant Responses in Pacific White Shrimp (*Litopenaeus vannamei*)

**DOI:** 10.3390/antiox15080968

**Published:** 2026-08-04

**Authors:** Thitirat Rattanawongwiboon, Natthapong Paankhao, Wararut Buncharoen, Benchawan Kumwan, Pakapon Meachasompop, Yosapon Adisornprasert, Chonlatat Rajitdumrong, Pimrawee Chaemlek, Prapansak Srisapoome, Kasinee Hemvichian, Passakorn Kingwascharapong, Anurak Uchuwittayakul

**Affiliations:** 1 Thailand Institute of Nuclear Technology (Public Organization), Ongkharak, Nakhon Nayok 26120, Thailand; thitirat@tint.or.th (T.R.); kasinee@tint.or.th (K.H.); 2 Kamphaeng Saen Fisheries Research Station, Faculty of Fisheries, Kasetsart University, Kamphaeng Saen Campus, Nakhon Pathom 73140, Thailand; ffisnpp@ku.ac.th; 3 Department of Biology, Faculty of Science, Chiang Mai University, Chiang Mai 50200, Thailand; wararut.bun@cmu.ac.th; 4 Department of Aquaculture, Faculty of Fisheries, Kasetsart University, Bangkok 10900, Thailand; benchawan.kumw@ku.th (B.K.); pakapon.meac@ku.th (P.M.); yosapon.ad@ku.th (Y.A.); chonlatat.ra@ku.th (C.R.); pimraee.ch@ku.th (P.C.); ffispssp@ku.ac.th (P.S.); 5 Laboratory of Aquatic Animal Health Management, Department of Aquaculture, Faculty of Fisheries, Kasetsart University, Bangkok 10900, Thailand; 6 Center of Excellence in Aquatic Animal Health Management, Faculty of Fisheries, Kasetsart University, Bangkok 10900, Thailand; 7 Department of Fishery Products, Faculty of Fisheries, Kasetsart University, Bangkok 10900, Thailand

**Keywords:** chitosan, low-molecular-weight chitosan, gamma irradiation, hydrogen peroxide, Pacific white shrimp, *Litopenaeus vannamei*, antioxidant response, innate immunity, *Vibrio parahaemolyticus*, disease resistance

## Abstract

This study evaluated the potential of synergistically degraded low-molecular-weight chitosan (LMW-CS) as a functional feed additive to promote growth, antioxidant capacity, innate immunity, and disease resistance in Pacific white shrimp (*Litopenaeus vannamei*). High-molecular-weight chitosan (HMW-CS, approximately 85 kDa) was degraded using γ-irradiation in combination with H_2_O_2_ to produce LMW-CS with improved functional properties. Shrimp were fed five experimental diets for 4 weeks: a control diet, HMW-CS0.4 (0.4% *w*/*w*), LMW-CS0.1 (0.1% *w*/*w*), LMW-CS0.2 (0.2% *w*/*w*), and LMW-CS0.4 (0.4% *w*/*w*). Growth performance, oxidative stress markers, antioxidant enzyme activities, lysozyme activity, immune-related gene expression, bacterial load, and survival after *Vibrio parahaemolyticus* challenge were evaluated. The results indicate that dietary LMW-CS supplementation improved growth performance and feed utilization, with LMW-CS0.2 showing significantly higher final weight, total weight gain, and average daily gain than the control group (*p* < 0.05). Antioxidant assays showed that LMW-CS reduced malondialdehyde levels and increased reduced glutathione, nitric oxide, glutathione reductase, catalase, superoxide dismutase, glutathione peroxidase, and glutathione-S-transferase activities in both plasma and hepatopancreas (*p* < 0.05). Lysozyme activity was significantly enhanced, particularly in the LMW-CS0.4 and HMW-CS0.4 groups (*p* < 0.05). Gene expression analysis revealed upregulation of genes associated with growth regulation, antimicrobial defense, pathogen recognition, and prophenoloxidase activation, including *igf2*, *cstn*, *lgbp*, *lyz*, and *propo2*. Gut microbiota profiling showed that chitosan supplementation altered bacterial community composition, reduced the relative abundance of some *Vibrio*-associated taxa, and descriptively lowered predicted pathogenic and stress-tolerant bacterial phenotypes. Following the *Vibrio parahaemolyticus* challenge, shrimp fed LMW-CS0.4 showed the lowest bacterial load and highest survival rate, indicating improved disease resistance (*p* < 0.05). Overall, synergistically degraded LMW-CS enhanced growth, redox balance, innate immune competence, gut microbial structure, and resistance to *V. parahaemolyticus*, supporting its potential as an antibiotic-free functional feed additive for sustainable shrimp aquaculture.

## 1. Introduction

Pacific white shrimp (*Litopenaeus vannamei*) is one of the most important farmed crustacean species worldwide and plays a central role in global shrimp aquaculture [1]. Its rapid growth, high market demand, good feed conversion, and adaptability to intensive farming systems have made it the dominant shrimp species in many producing countries, including Thailand [2,3]. However, the rapid intensification of shrimp farming has increased the risk of disease outbreaks, environmental stress, and oxidative imbalance, which together can reduce growth performance, impair immune competence, and cause severe economic losses [4]. Among the major bacterial diseases affecting shrimp production, acute hepatopancreatic necrosis disease (AHPND), commonly associated with pathogenic *Vibrio parahaemolyticus*, is considered one of the most destructive diseases in modern shrimp aquaculture [5,6,7].

AHPND mainly affects the hepatopancreas, an essential organ involved in digestion, nutrient absorption, metabolism, immune regulation, and antioxidant defense in shrimp. The disease is characterized by rapid onset of mortality, reduced feed intake, empty gut, pale or atrophied hepatopancreas, and severe histopathological damage to hepatopancreatic tubules [7,8]. Pathogenic AHPND-causing *V. parahaemolyticus* strains carry virulence genes encoding the *PirA* and *PirB* toxins, which are responsible for hepatopancreatic epithelial cell damage and disease progression [7,9]. Because shrimp lack a highly developed adaptive immune system, their defense against *Vibrio* infection depends largely on innate immune responses, including hemocyte activity, phenoloxidase activation, lysozyme-like activity, respiratory burst, antimicrobial peptides, and antioxidant defense systems. Therefore, nutritional strategies that strengthen innate immunity and redox homeostasis are highly important for improving shrimp resistance to AHPND [10].

The use of antibiotics and chemical agents to control bacterial diseases in shrimp farming has become increasingly restricted due to concerns over antimicrobial resistance, environmental contamination, residue accumulation, and consumer safety [11]. As a result, functional feed additives derived from natural, biodegradable, and immunologically active compounds have received increasing attention as sustainable alternatives for disease prevention. Among these candidates, chitosan is a promising biopolymer obtained by deacetylation of chitin, which is abundant in crustacean shell waste. Chitosan is biodegradable, biocompatible, non-toxic, and possesses several biological properties, including antimicrobial, antioxidant, immunostimulatory, and film-forming activities [12,13,14]. These characteristics make chitosan suitable for application in functional aquafeeds.

The biological activity of chitosan is strongly influenced by its molecular weight, degree of deacetylation, solubility, viscosity, and particle behavior. High-molecular-weight chitosan has useful structural and coating properties but often shows limited solubility and lower biological availability in aquatic animals. In contrast, low-molecular-weight chitosan and chitosan oligosaccharides generally exhibit improved solubility, lower viscosity, better dispersion, and stronger biological activities [15,16]. These properties may enhance interaction with the intestinal mucosa and improve the capacity of chitosan to modulate immune and antioxidant responses [17]. Therefore, reducing chitosan molecular weight without destroying its active amino and hydroxyl groups is an important strategy for improving its functional value as an aquafeed additive.

Synergistic degradation using gamma irradiation in combination with hydrogen peroxide is an effective approach for producing low-molecular-weight chitosan. Gamma irradiation can induce chain scission, while hydrogen peroxide enhances the formation of reactive hydroxyl radicals that accelerate depolymerization. This combined process can reduce molecular weight more efficiently than single degradation methods while preserving key functional groups responsible for biological activity. The resulting low-molecular-weight chitosan may have improved solubility, reduced viscosity, enhanced colloidal stability, and greater potential to act as an immunonutritional additive in shrimp diets.

Previous studies have shown that dietary chitosan-based additives can improve growth, immune responses, antioxidant status, intestinal health, and disease resistance in aquatic animals [18,19,20,21]. In shrimp, strengthening antioxidant defense is particularly important because *Vibrio* infection and intensive culture conditions can trigger oxidative stress, resulting in lipid peroxidation, tissue damage, immune suppression, and reduced survival [22]. Enhancement of antioxidant enzymes such as superoxide dismutase, catalase, glutathione peroxidase, and glutathione reductase, together with reduced malondialdehyde levels, may help maintain hepatopancreatic integrity during bacterial challenge. At the same time, stimulation of innate immune parameters, including total hemocyte count, phenoloxidase activity, respiratory burst, lysozyme-like activity, and antimicrobial-related gene expression, may improve shrimp resistance to *V. parahaemolyticus* infection [23].

Despite the promising potential of chitosan in aquaculture, limited information is available on the dietary application of synergistically degraded low-molecular-weight chitosan in Pacific white shrimp, particularly in relation to antioxidant responses and protection against AHPND caused by *V. parahaemolyticus*. Most previous studies have focused on conventional chitosan, chitosan nanoparticles, or chitosan conjugates, while the functional effects of chitosan produced through combined gamma irradiation and hydrogen peroxide degradation remain insufficiently understood in shrimp. In addition, the optimal inclusion level required to promote shrimp health without negatively affecting feed utilization or growth performance requires further investigation.

Therefore, the present study aimed to characterize synergistically degraded low-molecular-weight chitosan produced by gamma irradiation and hydrogen peroxide and to evaluate its dietary effects on health promotion, antioxidant responses, immune status, and disease resistance in Pacific white shrimp (*L. vannamei*) challenged with AHPND-causing *V. parahaemolyticus*. The study assessed the physicochemical properties of degraded chitosan, including molecular weight, viscosity, particle behavior, and functional stability, followed by an in vivo feeding trial to determine growth performance, feed utilization, hepatopancreatic antioxidant responses, innate immune parameters, immune-related gene expression, and survival after bacterial challenge. This work provides a sustainable approach for converting chitosan into a high-value functional feed additive and supports its potential application as an antibiotic-free strategy to improve shrimp health and resilience against AHPND in intensive aquaculture systems.

## 2. Materials and Methods

### 2.1. Development and Characterization of Synergistically Degraded Chitosan

#### 2.1.1. Materials

Chitosan (degree of deacetylation > 95%, molecular weight 500 kDa) was purchased from Bio21 Co., Ltd., (Chonburi, Thailand). Lactic acid (C_3_H_6_O_3_, 85%) was bought from Ajax Finechem Pty Ltd. (Taren Point, NSW, Australia). Hydrogen peroxide (H_2_O_2_, 30%) was acquired from Merck KGaA (Darmstadt, Germany). All other chemicals were of analytical reagent grade.

#### 2.1.2. Sample Preparation and γ-Irradiation Procedure

A chitosan solution was prepared by dissolving 15 g of chitosan in 400 mL of 1.5% (*w*/*v*) lactic acid under magnetic stirring until a homogeneous solution was obtained. Hydrogen peroxide was then added to obtain a final concentration of 0.5% (*w*/*v*). The resulting solution was irradiated using a ^60^Co gamma irradiator at the Thailand Institute of Nuclear Technology (Public Organization), Ongkharak, Nakhon Nayok, Thailand, at an absorbed dose of 10 kGy and a dose rate of 6.244 kGy/h under ambient conditions. Non-irradiated chitosan prepared under identical conditions, without the addition of H_2_O_2_ or gamma irradiation, was used as the control (conventional chitosan) for comparison throughout the study.

#### 2.1.3. Characterization of Molecular Weight by Gel Permeation Chromatography (GPC)

The molecular weight and polydispersity index (PDI) of the chitosan samples were determined by aqueous gel permeation chromatography (GPC). Prior to analysis, 20 mg of each dried sample was dissolved in 10 mL of 2% (*w*/*v*) aqueous acetic acid. Measurements were performed using a Shimadzu HPLC-based GPC system equipped with a refractive index (RI) detector and a Shodex SB-804 HQ column packed with polymeric polyether gel. Pullulan standards were employed for calibration. The mobile phase consisted of 0.2 M sodium acetate and was delivered at a flow rate of 0.5 mL/min. The column temperature was maintained at 30 °C, and 20 μL of each sample solution (2 mg/mL) was injected for analysis.

#### 2.1.4. Viscosity by Rheometer

The viscosity of the chitosan solutions was measured using a rheometer (HAAKE RheoStress, Thermo Scientific, Karlsruhe, Germany) at 25 °C under a constant shear rate of 100 s^−1^. Each sample was analyzed in five independent measurements, and the average value was reported.

### 2.2. Effects of Synergistically Degraded Low-Molecular-Weight Chitosan on Health Promotion, Antioxidant Responses, and Disease Resistance in Pacific White Shrimp (L. vannamei)

#### 2.2.1. Ethics Statement

All experimental procedures involving aquatic animals strictly followed the Ethical Principles and Guidelines for the Use of Animals established by the National Research Council of Thailand for scientific purposes. Additionally, the protocol received approval from the Animal Ethics Committee at Kasetsart University, Thailand (Approval No. ACKU67-FIS-017; approved on 20 May 2024).

#### 2.2.2. Animal Husbandry

Healthy Pacific white shrimp (*L. vannamei*) juveniles with an average body weight of approximately 1.0 ± 0.2 g were obtained from a commercial hatchery in Thailand. Upon arrival, shrimp were acclimated in 250 L fiberglass tanks containing aerated brackish water at a salinity of 10 ppt. During acclimation, water temperature was maintained at 28 ± 3 °C, dissolved oxygen was kept at ≥5 mg/L, and pH was maintained within the range of 7.5–8.2. Shrimp were acclimated for 14 days prior to the feeding trial to allow adaptation to laboratory conditions and the experimental salinity.

During the acclimation period, shrimp were stocked at an appropriate density and fed a commercial shrimp diet containing approximately 38–40% crude protein at 5% of body weight per day, divided into two daily feedings. Feed rations were adjusted according to observed feed intake and shrimp biomass during routine sampling. Uneaten feed, feces, and molts were removed daily to maintain water quality. Partial water exchange was performed regularly using dechlorinated brackish water adjusted to 10 ppt.

Routine health monitoring was conducted throughout the acclimation period. Shrimp were observed daily for survival, feeding activity, swimming behavior, molting condition, external abnormalities, and signs of disease. Moribund or dead shrimp were removed immediately and recorded. Before the start of the experiment, shrimp were randomly sampled to confirm the absence of gross clinical signs and bacterial infection. All husbandry procedures were conducted in accordance with institutional animal care and welfare guidelines to minimize stress and maintain optimal experimental conditions [24].

#### 2.2.3. Experimental Design

The two chitosan preparations used in this study consisted of native non-degraded chitosan with an approximate molecular weight of 85 kDa and synergistically depolymerized low-molecular-weight chitosan with an approximate molecular weight of 10 kDa. The low-molecular-weight chitosan was generated through γ-irradiation at 10 kGy in combination with 0.25% (*w*/*v*) H_2_O_2_. These chitosan preparations were incorporated into shrimp diets as functional feed additives, with inclusion levels selected according to their physicochemical characteristics and antibacterial activity profiles as previously described [21].

A four-week feeding experiment was carried out under a completely randomized design (CRD). The five experimental diets were randomly assigned to 20 shrimp tanks by drawing coded lots without replacement, with four replicate tanks allocated to each dietary treatment. Each tank contained 30 shrimp, giving a total of 120 shrimp per treatment. Juvenile Pacific white shrimp (*L. vannamei*) with an initial mean body weight of approximately 1.0 ± 0.2 g were randomly stocked into the tanks containing aerated brackish water adjusted to 10 ppt salinity.

The experimental diets were arranged as follows:

1. C: basal diet coated with sterile distilled water
2. HMW-CS0.4: basal diet supplemented with non-degraded high-molecular-weight chitosan at 0.4% *w*/*w*.
3. LMW-CS0.1: basal diet supplemented with degraded low-molecular-weight chitosan at 0.1% *w*/*w*.
4. LMW-CS0.2: basal diet supplemented with degraded low-molecular-weight chitosan at 0.2% *w*/*w*.
5. LMW-CS0.4: basal diet supplemented with degraded low-molecular-weight chitosan at 0.4% *w*/*w*.

For diet preparation, the respective chitosan coating solutions were evenly sprayed and mixed onto commercial shrimp feed pellets. The coated pellets were air-dried at room temperature for 30 min before use. Fresh experimental diets were prepared daily to maintain feed quality and coating stability. Shrimp were fed a commercial diet containing approximately 38–40% crude protein at 5% of body weight per day, divided into two equal meals, throughout the four-week trial. Feeding levels were adjusted weekly based on shrimp biomass and apparent feed consumption.

During the experimental period, salinity was maintained at 10 ppt. Water temperature was kept at 28 ± 3 °C, dissolved oxygen was maintained above 5 mg/L, and pH was maintained between 7.5 and 8.2. Uneaten feed, fecal matter, and molts were removed daily to preserve water quality. Partial water exchange was conducted routinely using dechlorinated brackish water prepared at the same salinity. Shrimp survival, feeding behavior, swimming activity, molting status, and general health condition were checked daily. Biomass in each tank was recorded weekly for feed adjustment.

At the designated sampling time, one shrimp was randomly selected from each of the four replicate tanks assigned to each dietary treatment, resulting in four shrimp per treatment (*n* = 4). Each shrimp was handled carefully and gently immobilized on ice to minimize movement and handling stress. Hemolymph was collected immediately from the ventral sinus using a sterile syringe, after which the shrimp was dissected aseptically to collect the target tissues. Plasma and tissue samples were processed or preserved immediately according to the requirements for immune-response, antioxidant, gene-expression, and microbiological analyses.

#### 2.2.4. Growth Performance Analysis

Shrimp were weighed at the beginning and end of the 4-week feeding trial to evaluate growth performance. At each sampling point, shrimp from each tank were gently collected, blotted dry with tissue paper, and weighed using a digital balance. The initial and final body weights were recorded to determine growth performance. Mortality was checked daily throughout the experiment, and the number of surviving shrimp was recorded at the end of the trial. Feed intake was recorded for each tank and used to calculate feed-utilization efficiency.

Total weight gain (TWG), average daily gain (ADG), specific growth rate (SGR), feed conversion ratio (FCR), and survival rate were calculated using the following equations:

1. TWG (g/shrimp) = final body weight − initial body weight
2. ADG (g/day) = (final body weight − initial body weight)/experimental period
3. SGR (%/day) = [(ln final body weight − ln initial body weight)/experimental period] × 100
4. FCR = total feed intake (g)/total weight gain (g)
5. Survival rate (%) = (final number of shrimp/initial number of shrimp) × 100

#### 2.2.5. Collection of Hemolymphs

At the designated sampling time, one shrimp was randomly selected from each of the four replicate tanks assigned to each dietary treatment. This yielded four independent biological replicates per treatment (*n* = 4), with each replicate originating from a separate tank. Each shrimp was handled carefully and gently immobilized on ice to minimize movement and handling stress. Hemolymph was collected immediately from the ventral sinus using a sterile syringe, after which the shrimp was dissected aseptically to collect the target tissues.

For immune and biochemical analyses, the remaining hemolymph was mixed with an equal volume of pre-chilled anticoagulant solution to prevent clotting. The hemolymph-anticoagulant mixture was centrifuged at 7500× *g* for 10 min at 4 °C to separate plasma from hemocytes. The plasma fraction was carefully collected and stored at −20 °C until further analysis of immune and antioxidant-related parameters.

For gene expression analysis, hemocytes and hepatopancreas were immediately transferred into RNA preservation reagent or directly processed for total RNA extraction using an RNeasy kit (Qiagen, Hilden, Germany), according to the manufacturer’s instructions. The extracted RNA was stored at −80 °C until subsequent quantitative real-time PCR analysis.

#### 2.2.6. qRT-PCR Analysis of Growth-, Antioxidant-, and Immune-Related Genes

Total RNA was extracted from hemocytes and hepatopancreas using the RNeasy kit (Qiagen, Hilden, Germany), following the manufacturer’s protocol. The concentration and purity of the extracted RNA were determined using a NanoDrop™ spectrophotometer (Thermo Fisher Scientific, Waltham, MA, USA). RNA samples with acceptable purity and integrity were selected for cDNA synthesis. First-strand cDNA was synthesized from 1 µg of total RNA using Maxime™ RT PreMix (iNtRON Biotechnology, Seongnam-si, Gyeonggi-do, Republic of Korea), according to the manufacturer’s instructions. The synthesized cDNA was stored at −20 °C until further analysis.

Quantitative real-time PCR was performed using the QuantiNova SYBR Green qPCR Kit (Qiagen, Hilden, Germany) with an AriaMx Real-Time PCR System (Agilent Technologies, Santa Clara, CA, USA). The amplification conditions consisted of an initial denaturation at 95 °C for 5 min, followed by 40 cycles of denaturation at 95 °C for 30 s, annealing at 60 °C for 30 s, and extension at 72 °C for 90 s. A final extension step was performed at 72 °C for 10 min. Melting curve analysis was included to verify amplification specificity.

The expression levels of growth-, antioxidant-, and immune-related genes were normalized using three reference genes, including *actb*, *ef1a*, and *gapdh*. Relative gene expression was calculated using the 2^−ΔΔCt^ method [25]. All primer pairs were validated for amplification efficiency and specificity before qRT-PCR analysis. The primer sequences of all target and reference genes are provided in Table 1.

#### 2.2.7. Measurement of Oxidative Stress Markers and Antioxidant Enzyme Activities in Plasma and Hepatopancrease

Oxidative stress markers and antioxidant enzyme activities were measured in plasma and hepatopancreas samples collected from Pacific white shrimp (*L. vannamei*) at the end of the 4-week feeding trial. Hemolymph was withdrawn from the ventral sinus using a sterile syringe containing pre-chilled anticoagulant solution and then centrifuged at 7500× *g* for 10 min at 4 °C. The plasma fraction was carefully collected and stored at −80 °C until biochemical analysis.

For hepatopancreas analysis, hepatopancreas tissues were aseptically dissected from the same sampled shrimp, gently rinsed with ice-cold physiological saline to remove residual hemolymph and external contaminants, blotted dry, and weighed. The tissues were homogenized in ice-cold phosphate-buffered saline or appropriate assay buffer at a ratio of 1:9 (*w*/*v*) using a tissue homogenizer. The homogenates were then centrifuged at 10,000× *g* for 15 min at 4 °C to remove cellular debris. The clear hepatopancreas homogenate supernatants were carefully collected and stored at −80 °C until analysis of oxidative stress markers and antioxidant enzyme activities.

(1) Thiobarbituric Acid Reactive Substances (TBARS) Assay

Lipid peroxidation was estimated by measuring malondialdehyde (MDA) levels using the TBARS method. Briefly, 0.1 mL of plasma or hepatopancreas homogenate supernatant was mixed with 0.2 mL of thiobarbituric acid solution and 1.0 mL of trichloroacetic acid, followed by the addition of an equal volume of 0.85% saline. The mixture was heated at 100 °C for 30 min and cooled to room temperature. Then, 2.0 mL of distilled water was added, and the mixture was centrifuged at 3500 rpm for 10 min. The absorbance of the clear supernatant was measured at 532 nm against a blank. MDA concentration was calculated using a tetramethoxypropane standard curve and expressed as µM MDA/mg protein.

(2) Reduced Glutathione (GSH) Content

Reduced glutathione levels were determined using a modified colorimetric method. Briefly, 1.0 mL of plasma or hepatopancreas homogenate supernatant was mixed with 1.0 mL of 4% sulfosalicylic acid and incubated at 4 °C for 1 h to precipitate proteins. After centrifugation at 3500 rpm for 20 min at 4 °C, the supernatant was mixed with 100 mM phosphate buffer of pH 7.4 and 100 mM 5,5′-dithiobis-(2-nitrobenzoic acid), also known as DTNB. The formation of the yellow-colored product was measured at 412 nm. GSH concentration was calculated using a GSH standard curve and expressed as mmol GSH/mg protein.

(3) Nitric Oxide (NO) Determination

Nitric oxide production was estimated by measuring nitrite concentration using the Griess reaction. Equal volumes of plasma or hepatopancreas homogenate supernatants and Griess reagent were mixed and incubated at room temperature. The absorbance of the resulting purple azo compound was measured at 546 nm. Nitrite concentration was calculated from a sodium nitrite standard curve and expressed as µM nitrite/mg protein.

(4) Glutathione Reductase (GR) Activity

Glutathione reductase activity was assessed by monitoring NADPH oxidation. Briefly, 0.1 mL of plasma or hepatopancreas homogenate supernatant was added to a reaction mixture containing 0.1 M phosphate buffer of pH 7.6, oxidized glutathione, and NADPH. The decrease in absorbance at 340 nm was recorded spectrophotometrically. GR activity was calculated using the extinction coefficient of NADPH and expressed as µM NADPH oxidized/min/mg protein.

(5) Catalase (CAT) Activity

Catalase activity was measured based on the decomposition of hydrogen peroxide. The reaction mixture consisted of 0.1 mL of plasma or hepatopancreas homogenate supernatants, 2.5 mL of 50 mM phosphate buffer of pH 5.0, and 0.4 mL of 5.9 mM H_2_O_2_. The decrease in absorbance was recorded at 240 nm every 30 s for 2 min. CAT activity was calculated and expressed as U/min/mg protein.

(6) Superoxide Dismutase (SOD) Activity

Superoxide dismutase activity was determined by measuring the inhibition of nitroblue tetrazolium reduction. Briefly, 0.1 mL of plasma or hepatopancreas homogenate supernatant was mixed with assay buffer containing 0.1 mM xanthine, 0.025 mM nitroblue tetrazolium, 0.1 mM EDTA, 60 mM sodium carbonate buffer, pH 10.2, and xanthine oxidase. The absorbance was measured at 560 nm. SOD activity was calculated using an SOD standard curve and expressed as U/min/mg protein.

(7) Glutathione Peroxidase (GPx) Activity

Glutathione peroxidase activity was measured by monitoring NADPH oxidation in the presence of glutathione reductase and reduced glutathione. The reaction mixture contained 0.1 M phosphate buffer of pH 7.4, 1 mM EDTA, 1 mM sodium azide, 1 U/mL glutathione reductase, 1 mM reduced glutathione, 0.2 mM NADPH, and 0.25 mM H_2_O_2_. The reaction was initiated by adding 0.1 mL of plasma or hepatopancreas homogenate supernatants, and the decrease in absorbance at 340 nm was recorded continuously for 3 min. GPx activity was calculated using the extinction coefficient of NADPH and expressed as mM NADPH oxidized/min/mg protein.

(8) Glutathione-S-Transferase (GST) Activity

Glutathione-S-transferase activity was determined using 1-chloro-2,4-dinitrobenzene, also known as CDNB, as the substrate. The reaction mixture contained 1.475 mL of 0.1 M phosphate buffer of pH 6.5, 0.2 mL of reduced glutathione, 0.025 mL of CDNB, and 0.3 mL of plasma or hepatopancreas homogenate supernatants. The formation of the GSH-CDNB conjugate was monitored at 340 nm. GST activity was calculated and expressed as µM conjugate formed/min/mg protein.

#### 2.2.8. Humoral Immune Response Assays

Lysozyme Activity

Lysozyme activity in shrimp plasma was determined using a turbidimetric assay based on the lysis of *Micrococcus lysodeikticus*, with slight modifications from the method of Parry et al. [28]. Briefly, *M. lysodeikticus* powder (Sigma-Aldrich, Darmstadt, Germany) was suspended in phosphate-buffered saline (PBS, pH 6.2) at a final concentration of 0.2 mg/mL. The bacterial suspension was prepared freshly and allowed to equilibrate at room temperature for 1 h before use.

For the assay, 10 µL of plasma sample was added to each well of a flat-bottom 96-well microplate, followed by 250 µL of the *M. lysodeikticus* suspension. The reaction mixture was incubated at room temperature, and absorbance was measured at 450 nm using a microplate spectrophotometer (iMark™ Microplate Absorbance Reader, BIO-RAD, Hercules, CA, USA). Absorbance readings were recorded at 0 and 5 min. A negative control containing PBS instead of plasma was included to correct for non-enzymatic changes in turbidity.

The rate of absorbance reduction was calculated as follows:

ΔA450/min = [(A0 − A5)/5]sample − [(A0 − A5)/5]negative control

Lysozyme activity was expressed as units per milliliter (U/mL), where one unit was defined as the amount of enzyme required to decrease absorbance by 0.001 per min under the assay conditions. The activity was calculated using the following equation:

Lysozyme activity (U/mL) = (ΔA450/min × *V*total)/(0.001 × *V*sample) × DF

where ΔA450/min is the change in absorbance per minute, *V*total is the total reaction volume (mL), *V*sample is the volume of plasma sample used in the reaction (mL), and DF is the dilution factor of the plasma sample.

#### 2.2.9. Gut Microbiota Profiling and Bioinformatics Analysis

To evaluate the effects of dietary chitosan supplementation on the intestinal bacterial communities of Pacific white shrimp, one shrimp was randomly collected from each of the four replicate tanks assigned to each dietary treatment at the end of the experiment, resulting in four independent intestinal samples per treatment (*n* = 4). Following aseptic dissection, intestinal contents were recovered under sterile conditions, rapidly snap-frozen in liquid nitrogen, and preserved at −80 °C until further processing.

Genomic DNA was isolated from intestinal material using the ZymoBIOMICS DNA Miniprep Kit (Zymo Research, Irvine, CA, USA) in accordance with the manufacturer’s protocol. DNA integrity was verified by 1.5% agarose gel electrophoresis, while concentration and purity were determined using a NanoDrop 2000 UV–Vis spectrophotometer (Thermo Fisher Scientific, Wilmington, DE, USA). The nearly full-length 16S rRNA gene (V1–V9 regions) was amplified with the universal primers 27F (AGRGTTTGATYNTGGCTCAG) and 1492R (TASGGHTACCTTGTTASGACTT), each appended with PacBio-compatible sample-specific barcodes to enable multiplexing. Polymerase chain reactions were performed using KOD One PCR Master Mix (Toyobo Life Science, Osaka, Japan) under the following conditions: an initial denaturation at 95 °C for 2 min; 25 amplification cycles consisting of denaturation at 98 °C for 10 s, annealing at 55 °C for 30 s, and extension at 72 °C for 90 s; and a terminal extension at 72 °C for 2 min.

Amplified products were purified with VAHTS DNA Clean Beads (Vazyme, Nanjing, China) and quantified using a Qubit dsDNA HS Assay Kit on a Qubit 3.0 Fluorometer (Invitrogen, Thermo Fisher Scientific, Carlsbad, CA, USA). Equimolar pooling of purified amplicons was followed by SMRTbell library preparation using the SMRTbell Express Template Prep Kit 2.0 (Pacific Biosciences, Menlo Park, CA, USA). Long-read sequencing was conducted on the PacBio Sequel II platform by Beijing Biomarker Technologies Co., Ltd. (Beijing, China) to generate circular consensus sequencing (CCS) reads.

Raw data were processed with SMRT Link software v8.0, retaining high-quality CCS reads with a minimum of five passes and a predicted accuracy of at least 0.90. Demultiplexing was carried out using lima v1.7.0 based on barcode information. Primer sequences were removed, and quality filtering was applied using Cutadapt v2.7 [29], and reads falling outside the expected length range (1200–1650 bp) were excluded. Chimeric sequences were detected and eliminated with the UCHIME algorithm v8.1 [30]. Subsequent denoising was performed using the DADA2 workflow to infer amplicon sequence variants (ASVs), providing single-nucleotide resolution of microbial diversity. ASVs represented by fewer than two total reads across all samples were discarded to minimize potential artifacts.

Taxonomic assignment of ASVs was conducted in QIIME2 using a Naïve Bayes classifier [31] trained on the SILVA rRNA reference database (release 138.1) [32] with a confidence cutoff of 70%. Within-sample diversity (alpha diversity) was assessed using Chao1, Shannon, and Simpson indices. Between-sample differences (beta diversity) were examined using distance-based metrics and visualized through principal coordinate analysis (PCoA) and non-metric multidimensional scaling (NMDS). Community similarity was further explored by hierarchical clustering using the UPGMA method, and relative taxonomic abundances were displayed as heatmaps.

Differentially abundant taxa associated with low-molecular-weight chitosan supplementation were identified using linear discriminant analysis effect size (LEfSe) [33], applying a logarithmic LDA score threshold of 4.0. In addition, redundancy analysis (RDA) implemented in the vegan package for R v.2.3 was used to investigate relationships between microbial community structure and experimental treatments. All sequence processing and statistical analyses were performed using the BMKCloud platform (https://www.biocloud.net).

#### 2.2.10. Disease Resistance, Bacterial Load, and Relative Percent Survival After *Vibrio parahaemolyticus* Challenge

At the end of the 4-week feeding trial, shrimp from each dietary treatment were used to evaluate disease resistance against AHPND-causing *Vibrio parahaemolyticus*. For each treatment, 80 shrimp were randomly selected, consisting of 20 shrimp from each of four replicate tanks, and transferred to 250 L fiberglass tanks containing aerated brackish water at 10 ppt salinity. Each treatment was maintained in four replicate tanks.

The pathogenic *Vibrio parahaemolyticus* strain was cultured in tryptic soy broth supplemented with 2% NaCl and incubated at 30 °C with shaking at 150 rpm until reaching the exponential growth phase. The bacterial suspension was then centrifuged at 6000× *g* for 10 min at 4 °C, washed twice with sterile 2% NaCl solution, and resuspended in sterile 2% NaCl solution to the required bacterial concentration for the challenge experiment. The bacterial concentration was adjusted to 1 × 10^7^ CFU/mL based on optical density and confirmed by plate counting. Each shrimp was challenged by intramuscular injection with 100 µL of the bacterial suspension, equivalent to 1 × 10^6^ CFU/shrimp. The challenge dose was selected based on a preliminary 14-day LD_50_ determination. Control shrimps were injected with sterile PBS.

After the challenge, shrimp were maintained under the same water conditions, including 10 ppt salinity, 28 ± 3 °C water temperature, dissolved oxygen ≥ 5 mg/L, and pH 7.5–8.2. Mortality and clinical signs were recorded daily for 14 days. Dead shrimp were removed immediately to prevent water deterioration and cross-contamination. Disease signs, including reduced feeding activity, lethargy, empty gut, pale hepatopancreas, and abnormal swimming behavior, were recorded when observed.

To determine the bacterial load after infection, hemolymph samples were aseptically collected from challenged shrimp at designated post-challenge time points. The hemolymph samples were serially diluted in sterile PBS, and 100 μL aliquots were spread onto TCBS selective agar for the detection of *Vibrio* spp. The plates were incubated at 30 °C for 24 h, after which bacterial colonies were counted. The bacterial load was expressed as CFU/mL of hemolymph.

Cumulative mortality was calculated for each treatment group during the 14-day observation period. Relative percent survival (RPS) was calculated using the following equation:

RPS (%) = [1 − (mortality rate of treated shrimp/mortality rate of control shrimp)] × 100

Disease resistance was evaluated based on cumulative mortality, RPS, clinical signs, and bacterial load in hemolymph after *V. parahaemolyticus* challenge.

#### 2.2.11. Statistical and Data Analysis

Growth performance, immune-related gene expression, antioxidant parameters, immune responses, bacterial load, and microbiota alpha-diversity indices are presented as mean ± SD. Before parametric analysis, data were assessed for normality using the Shapiro–Wilk test and for homogeneity of variances using the Brown–Forsythe test. Data satisfying these assumptions were analyzed using one-way ANOVA followed by Tukey’s multiple-comparison test. Statistical analyses and graphical presentations were performed using GraphPad Prism version 10.1.2 (GraphPad Software, San Diego, CA, USA). For the feeding trial, the tank was considered the independent experimental unit, with four replicate tanks per dietary treatment (*n* = 4). Growth and feed-utilization variables were calculated from tank-level records. For biochemical, immune, gene-expression, bacterial-load, and microbiota analyses, one shrimp was randomly sampled from each replicate tank, resulting in four independent biological replicates per treatment (*n* = 4); shrimp within a tank were not treated as independent replicates. Technical assay replicates were averaged before statistical analysis and were not treated as independent observations. Given the limited number of biological replicates, non-significant findings, and descriptive numerical patterns were interpreted cautiously. Survival after challenge with *Vibrio parahaemolyticus* was analyzed using Kaplan–Meier survival analysis followed by the log-rank (Mantel–Cox) test. Each challenge treatment comprised four replicate tanks containing 20 shrimp per tank, giving 80 shrimp per treatment. Differences were considered statistically significant at *p* < 0.05.

## 3. Results

### 3.1. Growth Performance

**Figure 1:**
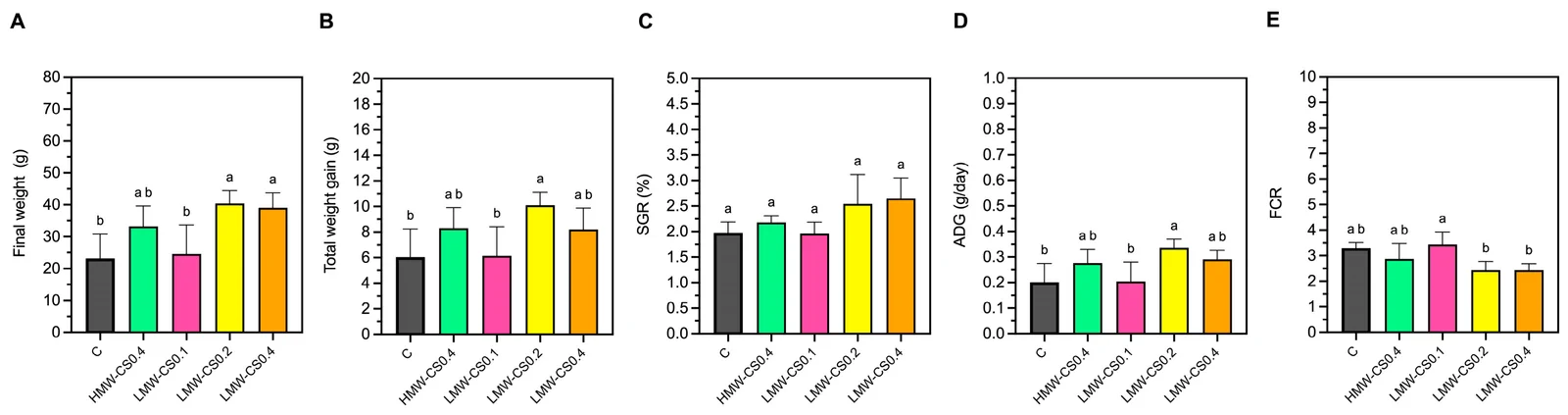
Effects of dietary high-molecular-weight chitosan and low-molecular-weight chitosan on growth performance of Pacific white shrimp (*L. vannamei*) after 4 weeks of feeding. (**A**) Final weight, (**B**) total weight gain, (**C**) SGR, (**D**) ADG, and (**E**) FCR of shrimp fed control, HMW-CS0.4, LMW-CS0.1, LMW-CS0.2, and LMW-CS0.4 diets. Data are presented as mean ± SD of four replicate tanks per treatment (*n* = 4 tanks). Growth and feed-utilization variables were calculated using tank-level records. Different lowercase letters indicate significant differences among treatments (*p* < 0.05).

The effects of dietary high-molecular-weight chitosan (HMW-CS) and low-molecular-weight chitosan (LMW-CS) on the growth performance of Pacific white shrimp are presented in Figure 1A–E. After the 4-week feeding trial, significant differences were observed among dietary treatments for final weight, total weight gain, average daily gain, specific growth rate, and feed conversion ratio.

Final weight was significantly affected by dietary treatment (*p* = 0.0050). Shrimp fed the LMW-CS0.2 and LMW-CS0.4 diets showed the highest final weights, with mean values of 40 ± 4.0 g and 39 ± 4.7 g, respectively, compared with the control group, which showed a mean final weight of 23 ± 7.6 g. The HMW-CS0.4 group showed an intermediate final weight of 33 ± 6.4 g, while the LMW-CS0.1 group showed a value similar to the control group at 25 ± 9.1 g.

Total weight gain was also significantly influenced by dietary chitosan supplementation (*p* = 0.0323). The highest total weight gain was recorded in the LMW-CS0.2 group at 10 ± 1.0 g, followed by the HMW-CS0.4 and LMW-CS0.4 groups at 8.3 ± 1.6 g and 8.2 ± 1.7 g, respectively. In contrast, the control and LMW-CS0.1 groups showed lower total weight gain values of 6.0 ± 2.2 g and 6.1 ± 2.3 g, respectively. The results indicated that the LMW-CS0.2 group was significantly higher than both the control and LMW-CS0.1 groups.

Specific growth rate (SGR) differed significantly among treatments (*p* = 0.0366). Although all groups showed positive growth throughout the trial, shrimp fed LMW-CS0.2 and LMW-CS0.4 tended to show higher SGR values than the control and LMW-CS0.1 groups. The mean SGR values were 2.5 ± 0.58% day^−1^ and 2.7 ± 0.40% day^−1^ in the LMW-CS0.2 and LMW-CS0.4 groups, respectively, compared with 2.0 ± 0.21% day^−1^ in the control group and 2.0 ± 0.23% day^−1^ in the LMW-CS0.1 group. However, the data showed no significant difference between individual treatment pairs, indicating that the overall treatment effect was modest.

Average daily gain (ADG) was significantly affected by dietary treatment (*p* = 0.0186). The highest ADG was observed in shrimp fed LMW-CS0.2 at 0.34 ± 0.033 g day^−1^, followed by LMW-CS0.4 at 0.29 ± 0.036 g day^−1^ and HMW-CS0.4 at 0.28 ± 0.053 g day^−1^. The control and LMW-CS0.1 groups showed lower ADG values of 0.20 ± 0.073 g day^−1^ and 0.20 ± 0.076 g day^−1^, respectively. The findings demonstrated that ADG in the LMW-CS0.2 group was significantly higher than that of the control and LMW-CS0.1 groups.

Feed conversion ratio (FCR) was significantly improved by dietary LMW-CS supplementation (*p* = 0.0068). The lowest FCR values were observed in the LMW-CS0.2 and LMW-CS0.4 groups, both with mean values of 2.4, indicating better feed-utilization efficiency. In contrast, the highest FCR was found in the LMW-CS0.1 group at 3.4 ± 0.49, followed by the control group at 3.3 ± 0.22. The data showed that the LMW-CS0.2 and LMW-CS0.4 groups had significantly lower FCR values than the LMW-CS0.1 group.

### 3.2. Lysozyme Activity

**Figure 2:**
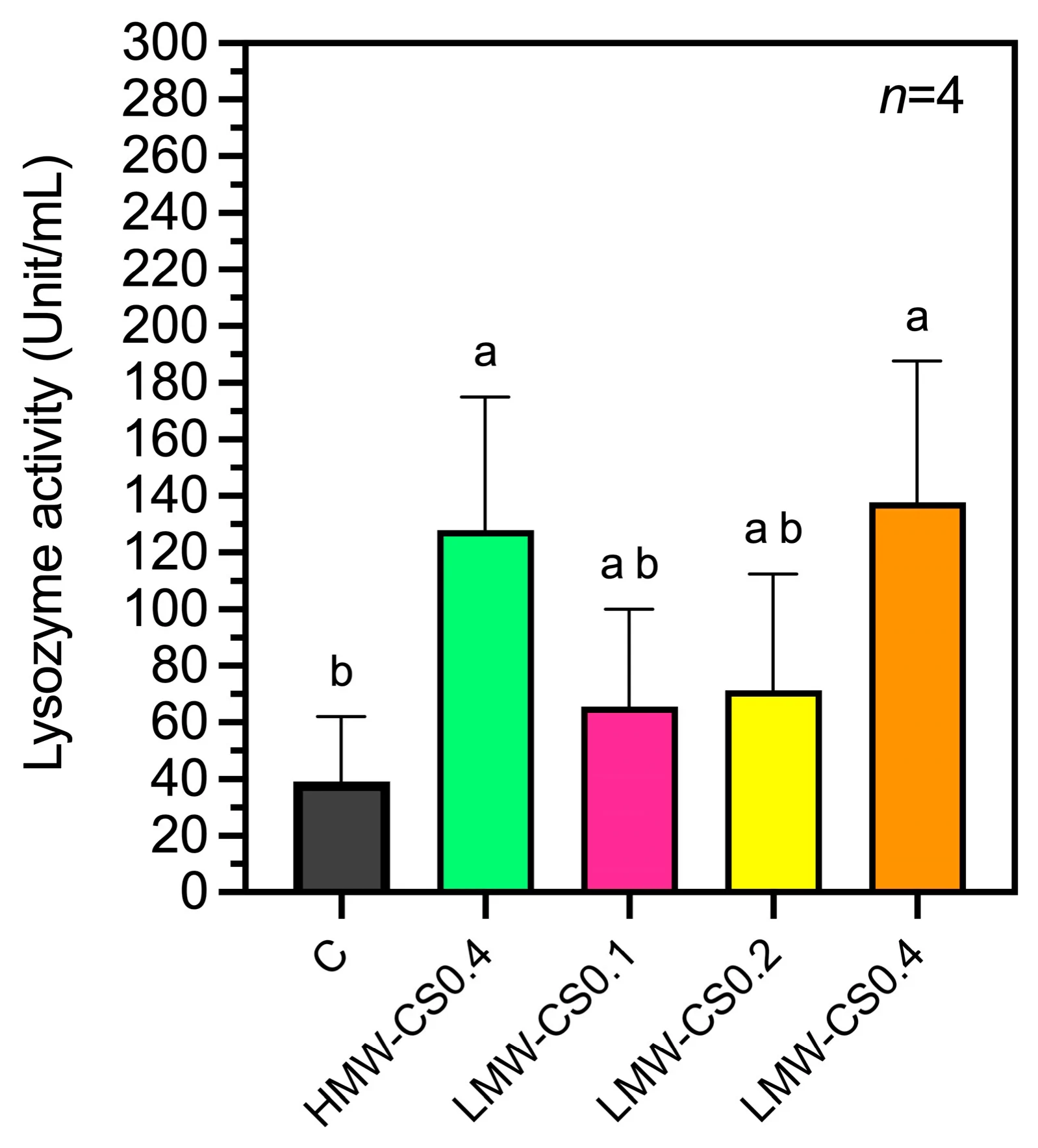
Effects of dietary chitosan on lysozyme activity in Pacific white shrimp (*L. vannamei*) fed control, HMW-CS0.4, LMW-CS0.1, LMW-CS0.2, and LMW-CS0.4 diets for 4 weeks. Data are presented as mean ± SD of four independent biological replicates per treatment (*n* = 4 shrimp, one shrimp sampled from each replicate tank). Different lowercase letters indicate significant differences among treatments (*p* < 0.05).

Dietary chitosan supplementation significantly enhanced lysozyme activity in Pacific white shrimp after 4 weeks of feeding (*p* = 0.0133). The highest lysozyme activities were observed in the LMW-CS0.4 and HMW-CS0.4 groups, with mean values of 138 ± 61 and 128 ± 33 U/mL, respectively. Both groups were significantly higher than the control group, which showed the lowest lysozyme activity at 39 ± 18 U/mL. The LMW-CS0.1 and LMW-CS0.2 groups showed intermediate values of 66 ± 43 and 71 ± 31 U/mL, respectively (Figure 2).

### 3.3. Gene Expression Profiles in Hemocytes and Hepatopancreas

**Figure 3:**
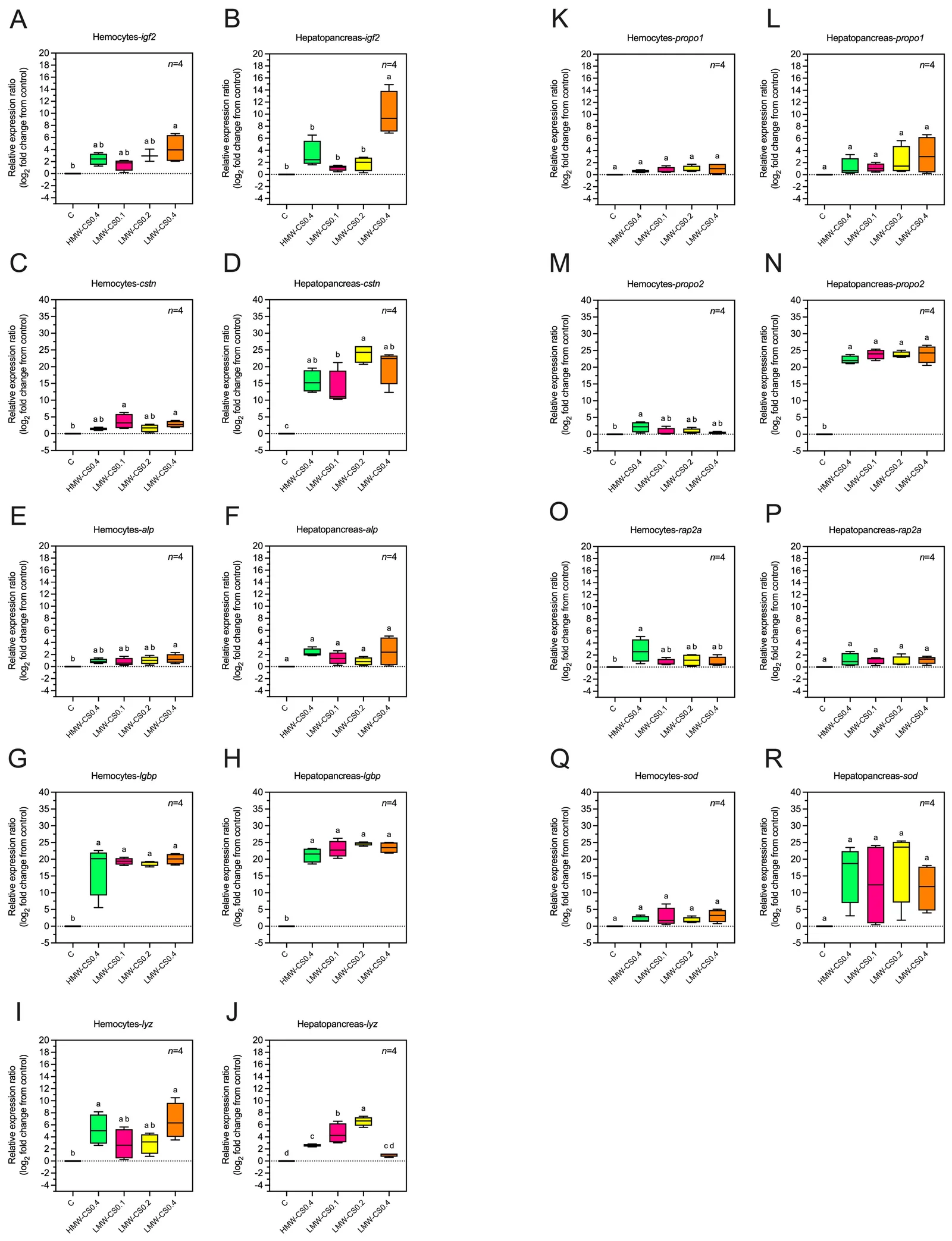
Effects of dietary chitosan on gene expression profiles in hemocytes and hepatopancreas of Pacific white shrimp (*Litopenaeus vannamei*). Relative expression levels of growth-, immune-, and antioxidant-related genes were determined in hemocytes and hepatopancreas after 4 weeks of feeding with the control, HMW-CS0.4, LMW-CS0.1, LMW-CS0.2, and LMW-CS0.4 diets. Panels show the expression of insulin-like growth factor 2 (*igf2*) in hemocytes (**A**) and hepatopancreas (**B**), crustin-like antimicrobial peptide (*cstn*) in hemocytes (**C**) and hepatopancreas (**D**), alkaline phosphatase (*alp*) in hemocytes (**E**) and hepatopancreas (**F**), lipopolysaccharide- and β-1,3-glucan-binding protein (*lgbp*) in hemocytes (**G**) and hepatopancreas (**H**), lysozyme (*lyz*) in hemocytes (**I**) and hepatopancreas (**J**), prophenoloxidase 1 (*propo1*) in hemocytes (**K**) and hepatopancreas (**L**), prophenoloxidase 2 (*propo2*) in hemocytes (**M**) and hepatopancreas (**N**), Ras-related protein Rap-2a (*rap2a*) in hemocytes (**O**) and hepatopancreas (**P**), and superoxide dismutase (*sod*) in hemocytes (**Q**) and hepatopancreas (**R**). Data are expressed as relative expression ratio and log_2_ fold change from the control. Data are presented as mean ± SD of four independent biological replicates per treatment (*n* = 4 shrimp, one shrimp sampled from each replicate tank). Different lowercase letters indicate significant differences among dietary treatments (*p* < 0.05).

The relative expression levels of growth-, immune-, and antioxidant-related genes in hemocytes and hepatopancreas of Pacific white shrimp are shown in Figure 3A–R. Dietary chitosan supplementation significantly modulated several gene transcripts, with tissue-specific expression patterns observed between hemocytes and hepatopancreas.

In hemocytes, *igf2* expression was significantly upregulated by dietary chitosan supplementation (Figure 3A). The highest expression was observed in the LMW-CS0.4 group, which was significantly higher than the control group (*p* < 0.05), whereas the HMW-CS0.4, LMW-CS0.1, and LMW-CS0.2 groups did not differ significantly from the control group (*p* > 0.05). In the hepatopancreas, *igf2* expression was markedly increased in the LMW-CS0.4 group, which was significantly higher than all other groups (Figure 3B; *p* < 0.05).

The antimicrobial peptide gene *cstn* was also affected by dietary treatment. In hemocytes, *cstn* expression was significantly increased in the LMW-CS0.1 and LMW-CS0.4 groups compared with the control group (Figure 3C; *p* < 0.05). In the hepatopancreas, *cstn* expression was strongly upregulated in all chitosan-supplemented groups compared with the control, with the highest expression observed in the LMW-CS0.2 group (Figure 3D; *p* < 0.05).

For *alp*, hemocyte expression was significantly increased in the LMW-CS0.4 group compared with the control group (Figure 3E; *p* < 0.05), while the HMW-CS0.4, LMW-CS0.1, and LMW-CS0.2 groups remained comparable to that in the control (*p* > 0.05). In contrast, hepatopancreatic *alp* expression did not differ significantly among treatments (Figure 3F; *p* > 0.05).

The pathogen-recognition gene *lgbp* was strongly induced by dietary chitosan. In hemocytes, all chitosan-supplemented groups showed significantly higher *lgbp* expression than the control group (Figure 3G; *p* < 0.05). A similar pattern was observed in the hepatopancreas, where all chitosan-fed groups showed significant upregulation of *lgbp* compared with the control group (Figure 3H; *p* < 0.05).

Hemocyte *lyz* expression was significantly enhanced by dietary chitosan supplementation (Figure 3I). The HMW-CS0.4 and LMW-CS0.4 groups showed the highest expression level and were significantly higher than the control group (*p* < 0.05), whereas expression in the LMW-CS0.1 and LMW-CS0.2 groups remained comparable to that in the control group (*p* > 0.05). In the hepatopancreas, *lyz* expression differed significantly among treatments (Figure 3J; *p* < 0.05). The highest expression was observed in the LMW-CS0.2 group, followed by LMW-CS0.1 and HMW-CS0.4, whereas the LMW-CS0.4 group did not differ significantly from that in the control group (*p* > 0.05).

The expression of *propo1* did not differ significantly among dietary treatments in hemocytes (Figure 3K; *p* > 0.05) or hepatopancreas (Figure 3L; *p* > 0.05), despite a slight increasing trend in some chitosan-supplemented groups.

For *propo2*, hemocyte expression was significantly higher only in the HMW-CS0.4 group than in the control group (Figure 3M; *p* < 0.05). In contrast, hepatopancreatic *propo2* expression was significantly upregulated in all chitosan-fed groups compared with the control group (Figure 3N; *p* < 0.05).

Hemocyte *rap2a* expression was significantly increased in the HMW-CS0.4 group compared with the control group (Figure 3O; *p* < 0.05), whereas all LMW-CS groups remained comparable to the control group (*p* > 0.05). Hepatopancreatic *rap2a* expression did not differ significantly among treatments (Figure 3P; *p* > 0.05).

The antioxidant-related gene *sod* showed no significant differences among treatments in hemocytes (Figure 3Q; *p* > 0.05). Similarly, hepatopancreatic *sod* expression was statistically comparable among groups despite high variability among chitosan-fed shrimp (Figure 3R; *p* > 0.05).

Overall, the results indicate that dietary chitosan supplementation, particularly LMW-CS0.2 and LMW-CS0.4, significantly enhanced the transcriptional responses of genes associated with growth regulation, antimicrobial defense, pathogen recognition, and prophenoloxidase activation. The most prominent responses were observed for *igf2*, *cstn*, *lgbp*, *lyz*, and *propo2*.

### 3.4. Oxidative Stress Markers and Antioxidant Enzyme Activities in Plasma and Hepatopancreas

**Figure 4:**
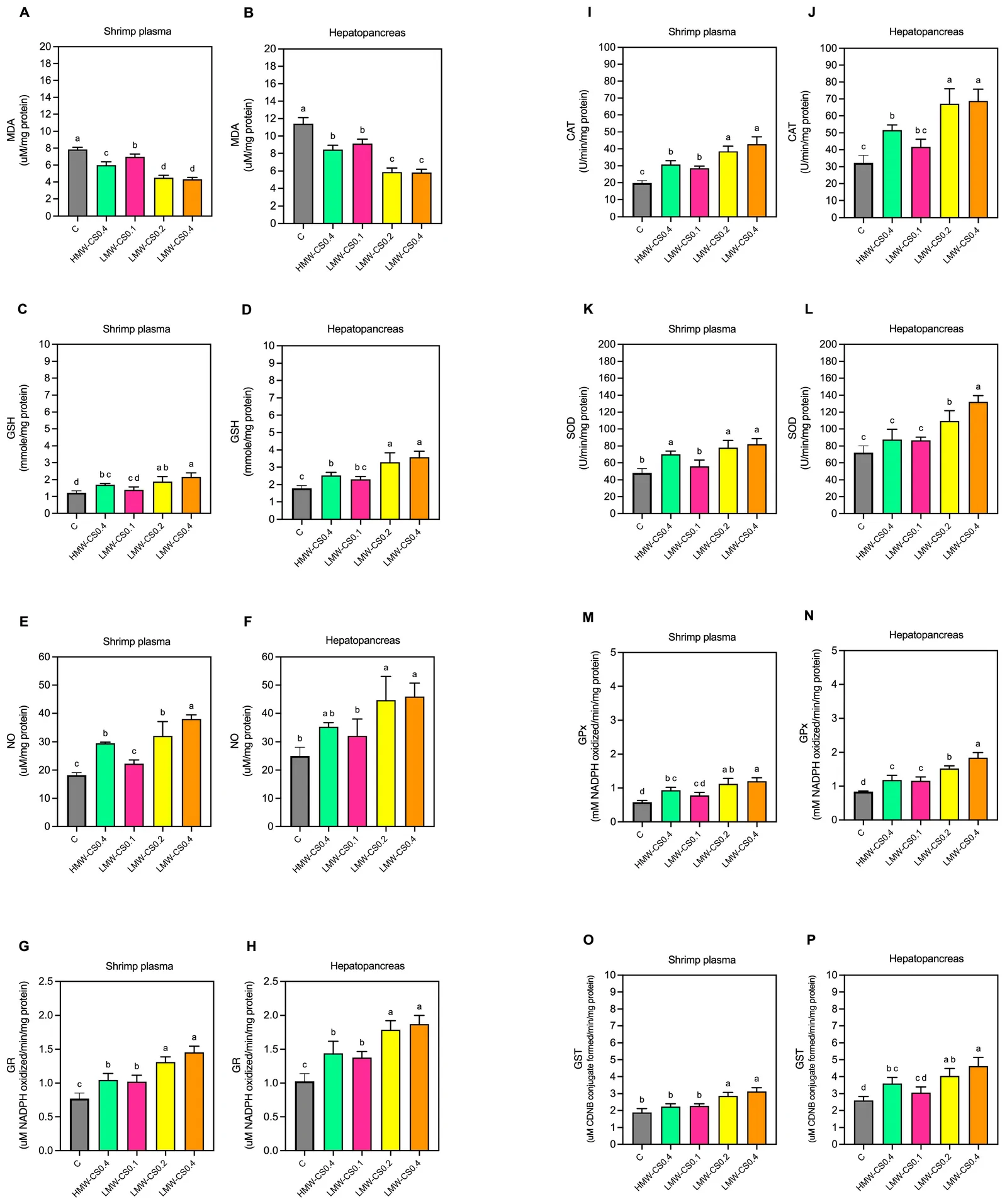
Effects of dietary chitosan on oxidative stress markers and antioxidant enzyme activities in plasma and hepatopancreas of Pacific white shrimp (*L. vannamei*). Shrimp were fed control, HMW-CS0.4, LMW-CS0.1, LMW-CS0.2, or LMW-CS0.4 diets for 4 weeks. Oxidative stress and antioxidant parameters were measured in plasma and hepatopancreas, including MDA in plasma (**A**) and hepatopancreas (**B**), GSH in plasma (**C**) and hepatopancreas (**D**), NO in plasma (**E**) and hepatopancreas (**F**), GR in plasma (**G**) and hepatopancreas (**H**), CAT in plasma (**I**) and hepatopancreas (**J**), SOD in plasma (**K**) and hepatopancreas (**L**), GPx in plasma (**M**) and hepatopancreas (**N**), and GST in plasma (**O**) and hepatopancreas (**P**). Data are presented as mean ± SD of four independent biological replicates per treatment (*n* = 4 shrimp, one shrimp sampled from each replicate tank). Different lowercase letters indicate significant differences among treatments (*p* < 0.05).

The effects of dietary chitosan supplementation on oxidative stress markers and antioxidant enzyme activities in plasma and hepatopancreas of Pacific white shrimp are shown in Figure 4A–P. Significant differences were observed among dietary treatments for all measured parameters, including MDA, GSH, NO, GR, CAT, SOD, GPx, and GST.

In shrimp plasma, MDA levels were significantly reduced in shrimp fed chitosan-supplemented diets compared with the control group (Figure 4A; *p* < 0.05). The lowest MDA levels were observed in the LMW-CS0.2 and LMW-CS0.4 groups, indicating reduced lipid peroxidation. A similar pattern was observed in the hepatopancreas, where dietary chitosan significantly decreased MDA levels compared with the control group (Figure 4B; *p* < 0.05). The LMW-CS0.2 and LMW-CS0.4 groups showed the strongest reduction in hepatopancreatic MDA.

Plasma GSH content was significantly increased by dietary chitosan supplementation (Figure 4C; *p* < 0.05). The highest GSH level was detected in the LMW-CS0.4 group, followed by LMW-CS0.2, while the control group showed the lowest value. Hepatopancreatic GSH content also increased significantly in chitosan-fed shrimp (Figure 4D; *p* < 0.05), with LMW-CS0.2 and LMW-CS0.4 showing the highest levels among the treatments.

Nitric oxide production in plasma was significantly enhanced by dietary chitosan supplementation (Figure 4E; *p* < 0.05). The LMW-CS0.4 group showed the highest NO level, followed by LMW-CS0.2, whereas the control and HMW-CS0.4 groups showed lower values. In the hepatopancreas, NO levels were also significantly increased in shrimp fed chitosan-supplemented diets (Figure 4F; *p* < 0.05), with the highest values observed in the LMW-CS0.2 and LMW-CS0.4 groups.

Plasma GR activity was significantly elevated in the chitosan-supplemented groups compared with the control group (Figure 4G; *p* < 0.05). The highest GR activities were observed in the LMW-CS0.2 and LMW-CS0.4 groups. A similar response was found in the hepatopancreas, where GR activity was significantly increased by dietary chitosan supplementation (Figure 4H; *p* < 0.05). The LMW-CS0.2 and LMW-CS0.4 groups showed the greatest enhancement.

Catalase activity in plasma was significantly increased in shrimp fed chitosan-supplemented diets (Figure 4I; *p* < 0.05). The LMW-CS0.2 and LMW-CS0.4 groups showed the highest CAT activities, while the control group showed the lowest activity. Hepatopancreatic CAT activity showed a similar pattern (Figure 4J; *p* < 0.05), with significantly higher activity in the LMW-CS0.2 and LMW-CS0.4 groups than in the control group.

Plasma SOD activity was significantly enhanced by dietary chitosan supplementation (Figure 4K; *p* < 0.05). The LMW-CS0.1, LMW-CS0.2, and LMW-CS0.4 groups showed higher SOD activities than the control and HMW-CS0.4 groups. In the hepatopancreas, SOD activity was also significantly increased among treatments (Figure 4L; *p* < 0.05). The highest activity was observed in the LMW-CS0.4 group, followed by LMW-CS0.2.

Plasma GPx activity was significantly increased in response to chitosan supplementation (Figure 4M; *p* < 0.05). The LMW-CS0.4 group showed the highest GPx activity, followed by LMW-CS0.2, whereas the control group showed the lowest value. Hepatopancreatic GPx activity was also significantly elevated in chitosan-fed shrimp (Figure 4N; *p* < 0.05), with the highest activity observed in the LMW-CS0.4 group.

GST activity in plasma was significantly enhanced by dietary chitosan supplementation (Figure 4O; *p* < 0.05). The LMW-CS0.2 and LMW-CS0.4 groups showed the highest GST activities, indicating improved detoxification-related antioxidant capacity. In the hepatopancreas, GST activity also differed significantly among treatments (Figure 4P; *p* < 0.05), with the highest activity observed in the LMW-CS0.4 group, followed by LMW-CS0.2.

### 3.5. Microbial Community Composition and Structure

**Figure 5:**
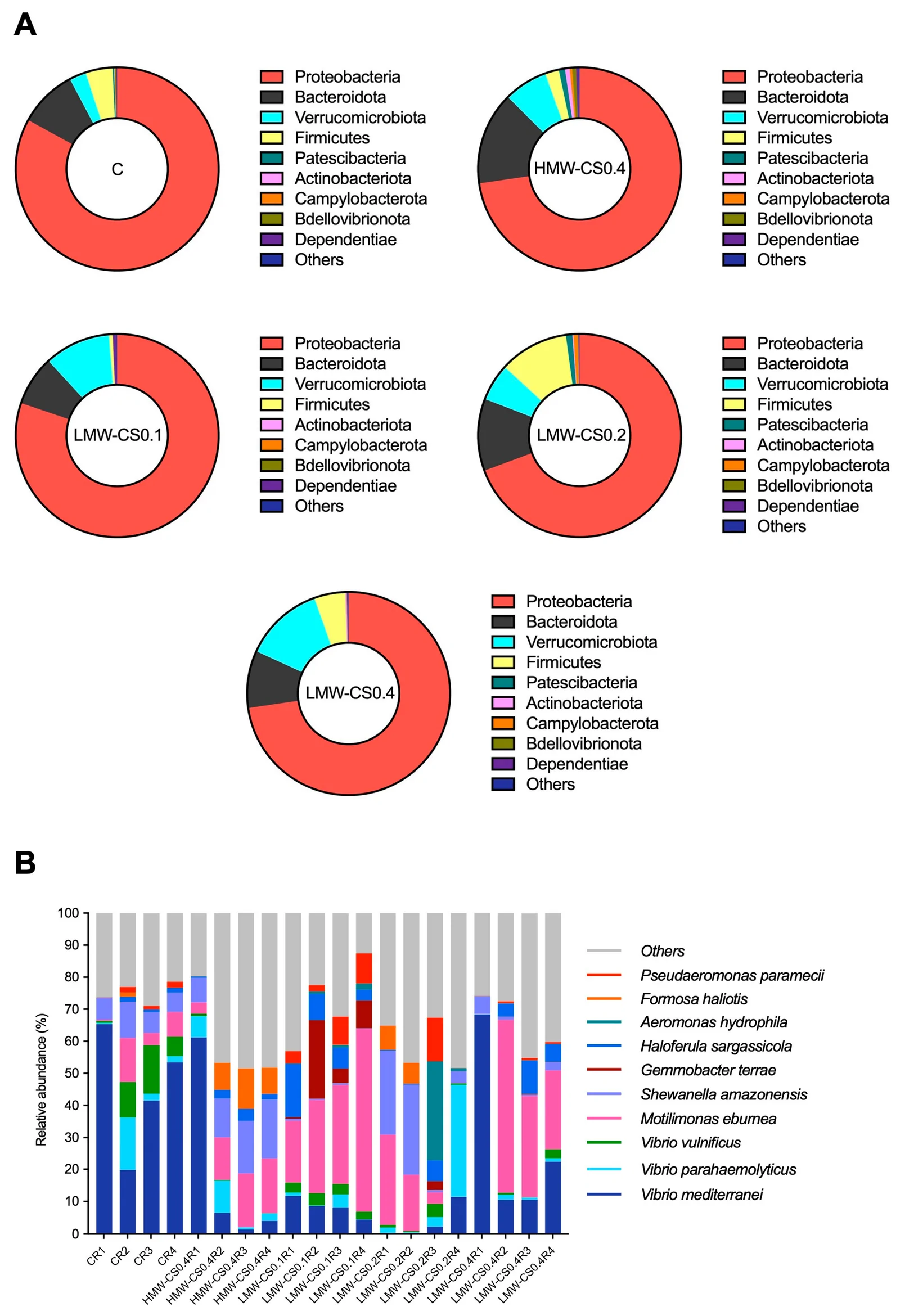
Effects of dietary chitosan on bacterial community composition in Pacific white shrimp (*L. vannamei*) fed control, HMW-CS0.4, LMW-CS0.1, LMW-CS0.2, and LMW-CS0.4 diets for 4 weeks. (**A**) Relative abundance of bacterial phyla among treatment groups. (**B**) Relative abundance of dominant bacterial species across individual samples. Data are shown as relative abundance (%). Four independent intestinal samples were analyzed per treatment (*n* = 4 shrimp, one shrimp sampled from each replicate tank). These relative-abundance patterns are descriptive and should not be interpreted as statistically confirmed treatment differences.

At the phylum level, Proteobacteria dominated the bacterial community in all groups, ranging from 69.36% in LMW-CS0.2 to 82.97% in the control group. Bacteroidota was the second most abundant phylum, with the highest proportion in HMW-CS0.4 (14.80%) and the lowest in LMW-CS0.1 (7.97%). Verrucomicrobiota increased in the chitosan-treated groups, especially in LMW-CS0.4 (12.69%) and LMW-CS0.1 (10.59%), compared with the control group (2.69%). Firmicutes was particularly enriched in LMW-CS0.2 (11.03%), while remaining lower in the other groups. Minor phyla, including Patescibacteria, Actinobacteriota, Campylobacterota, Bdellovibrionota, and Dependentiae, were present at low relative abundances, generally below 1%. Overall, the phylum-level profile indicated that Proteobacteria remained the dominant bacterial group across all treatments, whereas chitosan supplementation was associated with shifts in the relative proportions of Bacteroidota, Verrucomicrobiota, and Firmicutes. These relative-abundance patterns are descriptive and should not be interpreted as statistically confirmed treatment differences (Figure 5A).

At the species level, the bacterial community displayed clear compositional differences among the experimental groups. *Vibrio mediterranei* was the dominant species in the control group (45.03%) and remained relatively abundant in LMW-CS0.4 (27.96%) and HMW-CS0.4 (18.30%), whereas its abundance decreased markedly in LMW-CS0.1 (8.24%) and LMW-CS0.2 (3.55%). Other *Vibrio* species also varied among treatments, with *V. parahaemolyticus* reaching its highest abundance in LMW-CS0.2 (10.06%) and *V. vulnificus* being most abundant in the control group (8.21%) but reduced in the chitosan-treated groups. In contrast, *Motilimonas eburnea* increased markedly in the chitosan treatments, particularly in LMW-CS0.1 (34.06%) and LMW-CS0.4 (27.61%), compared with the control group (6.37%). *Shewanella amazonensis* was enriched in LMW-CS0.2 (14.70%) and HMW-CS0.4 (13.77%), while *Gemmobacter terrae* and *Haloferula sargassicola* were most abundant in LMW-CS0.1, accounting for 9.58% and 8.65%, respectively. *Aeromonas hydrophila* was nearly absent in the control and HMW-CS0.4 groups but increased in LMW-CS0.2 (7.99%), mainly due to its high abundance in one replicate. *Formosa haliotis* was most abundant in HMW-CS0.4 (7.29%), whereas *Pseudaeromonas paramecii* was enriched in LMW-CS0.1 (6.02%) and LMW-CS0.2 (3.44%). The “Others” category accounted for 24.92%, 40.76%, 27.58%, 40.69%, and 34.72% of the bacterial community in the control, HMW-CS0.4, LMW-CS0.1, LMW-CS0.2, and LMW-CS0.4 groups, respectively. These taxonomic profiles are presented descriptively and do not establish statistically significant treatment effects (Figure 5B).

### 3.6. Differential Alpha Diversity Analysis

**Figure 6:**
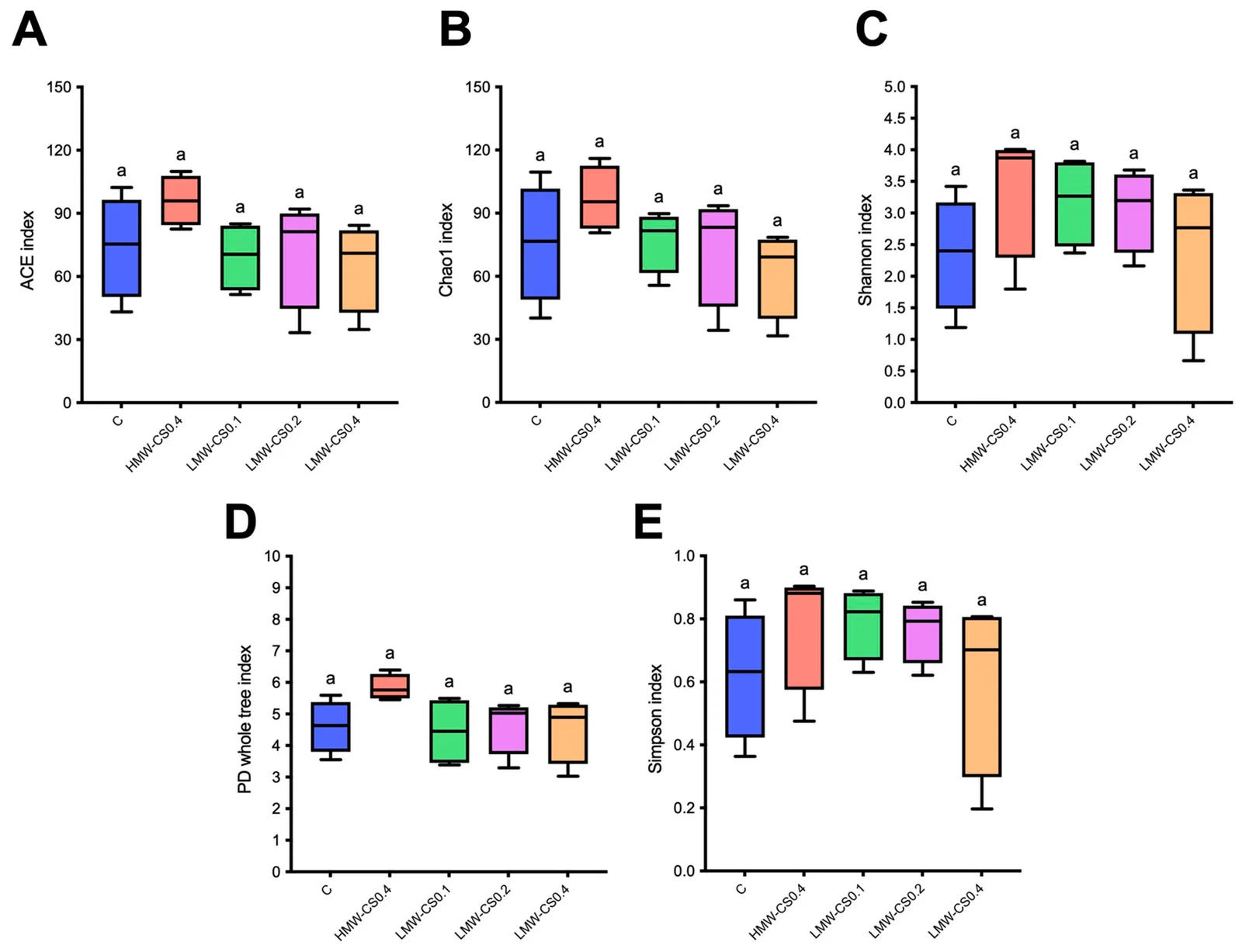
Effects of dietary chitosan on bacterial alpha diversity in Pacific white shrimp (*L. vannamei*) fed control, HMW-CS0.4, LMW-CS0.1, LMW-CS0.2, and LMW-CS0.4 diets for 4 weeks. (**A**) ACE index, (**B**) Chao1 index, (**C**) Shannon index, (**D**) PD whole tree index, and (**E**) Simpson index. Data are shown as box plots. Four independent intestinal samples were analyzed per treatment (*n* = 4 shrimp, one shrimp sampled from each replicate tank). Different letters above bars indicate statistically significant differences among groups (*p* < 0.05).

Alpha diversity analysis showed no significant differences among the experimental groups for all evaluated indices, including ACE, Chao1, Simpson, Shannon, and PD whole tree. Although the HMW-CS0.4 group showed numerically higher richness estimates, with ACE and Chao1 values of 96.04 ± 12.09 and 96.91 ± 15.57, respectively, these differences were not statistically significant among groups (ACE, *p* = 0.3026; Chao1, *p* = 0.3173). Similarly, Simpson and Shannon diversity indices showed no significant differences among treatments (Simpson, *p* = 0.4847; Shannon, *p* = 0.4345), although HMW-CS0.4 and LMW-CS0.1 displayed numerically higher diversity values than the control group. Phylogenetic diversity, represented by PD whole tree, also did not differ significantly among groups (*p* = 0.2076), despite the highest numerical value being observed in HMW-CS0.4. These results indicate that supplementation with high- or low-molecular-weight chitosan at different concentrations did not significantly alter bacterial alpha diversity, although a numerical trend toward increased richness and diversity was observed in the HMW-CS0.4 group. Accordingly, the observed numerical variation among group means was not interpreted as evidence of treatment-related changes in bacterial richness or diversity (Figure 6A–E).

### 3.7. Beta Diversity

#### 3.7.1. Principal Coordinate Analysis (PCoA)

**Figure 7:**
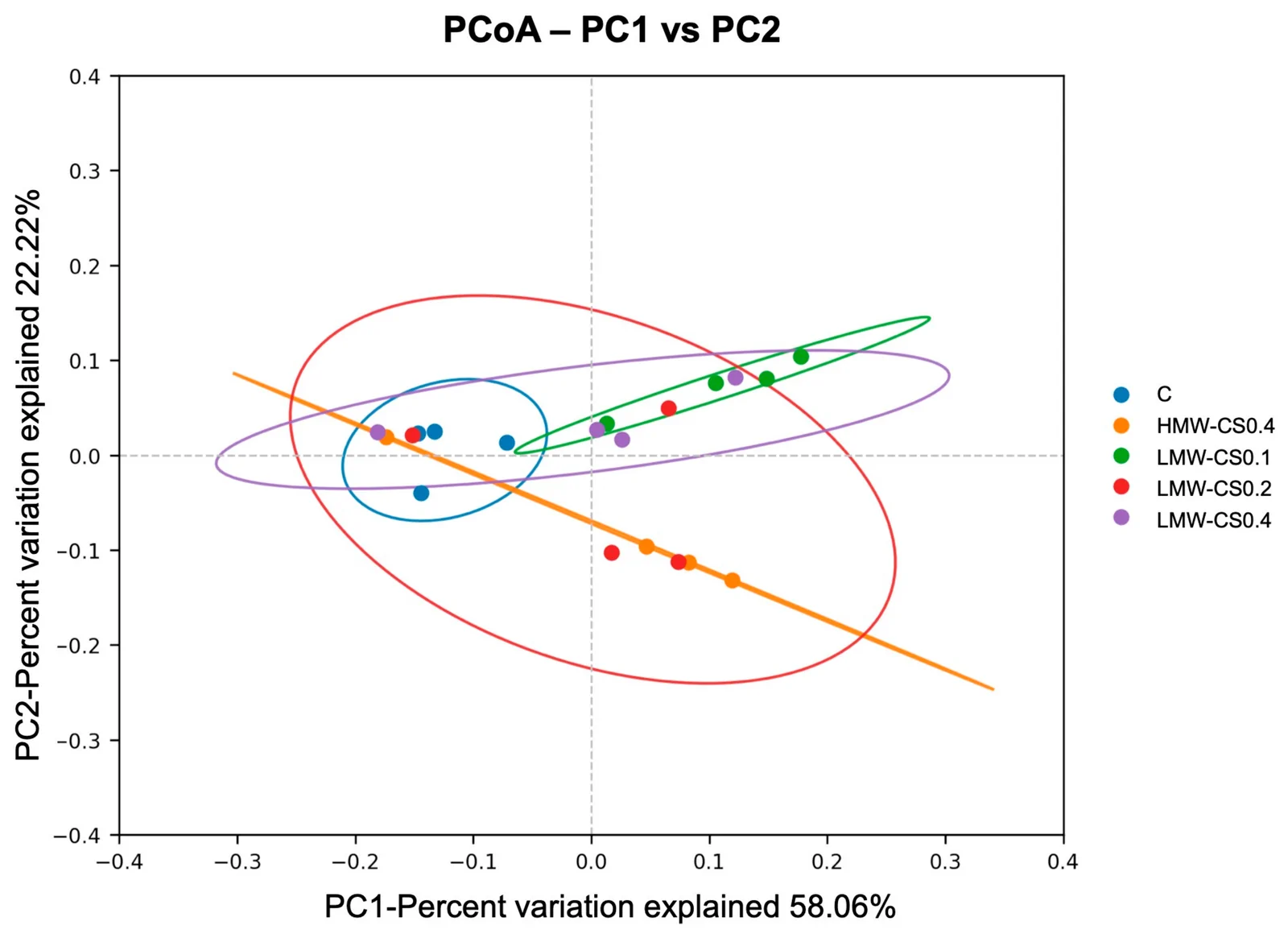
Weighted UniFrac-based principal coordinate analysis (PCoA) of bacterial communities in Pacific white shrimp (*L. vannamei*) fed control, HMW-CS0.4, LMW-CS0.1, LMW-CS0.2, and LMW-CS0.4 diets for 4 weeks. PC1 and PC2 explained 58.06% and 22.22% of the total variation, respectively. Points represent individual samples, and ellipses indicate the distribution of samples within each treatment group. Four independent intestinal samples were analyzed per treatment (*n* = 4 shrimp, one shrimp sampled from each replicate tank).

PCoA based on weighted UniFrac distance was used to visualize differences in the phylogenetically weighted bacterial community structure among dietary treatments. The first two principal coordinates explained 80.28% of the total variation, with PC1 and PC2 accounting for 58.06% and 22.22%, respectively. The control group was mainly positioned on the negative side of PC1 and clustered relatively close to the origin, indicating a community profile that differed from several chitosan-treated groups. The HMW-CS0.4 group, which received a basal diet supplemented with non-degraded high-molecular-weight chitosan at 0.4% *w*/*w*, showed a wider distribution along PC1 and PC2, with one replicate located near the control cluster and the remaining replicates shifted toward the positive PC1 and negative PC2 regions. In contrast, LMW-CS0.1, representing a basal diet supplemented with degraded low-molecular-weight chitosan at 0.1% *w*/*w*, formed a more distinct cluster on the positive side of both PC1 and PC2, suggesting a clear shift in bacterial community composition relative to the control. The LMW-CS0.2 group showed the greatest dispersion, with samples distributed across both negative and positive PC1 values, indicating higher within-group variation. The LMW-CS0.4 group was positioned mainly around the central to positive PC1 region and partially overlapped with other treatment groups. Overall, samples showed partial separation as well as overlap among treatment groups, and dispersion varied within several groups, particularly HMW-CS0.4 and LMW-CS0.2. Because no inferential test of beta-diversity differences is reported here, the ordination pattern is interpreted descriptively and does not establish a statistically significant treatment effect on community structure (Figure 7).

#### 3.7.2. UPGMA Clustering Tree with Taxonomic Composition

**Figure 8:**
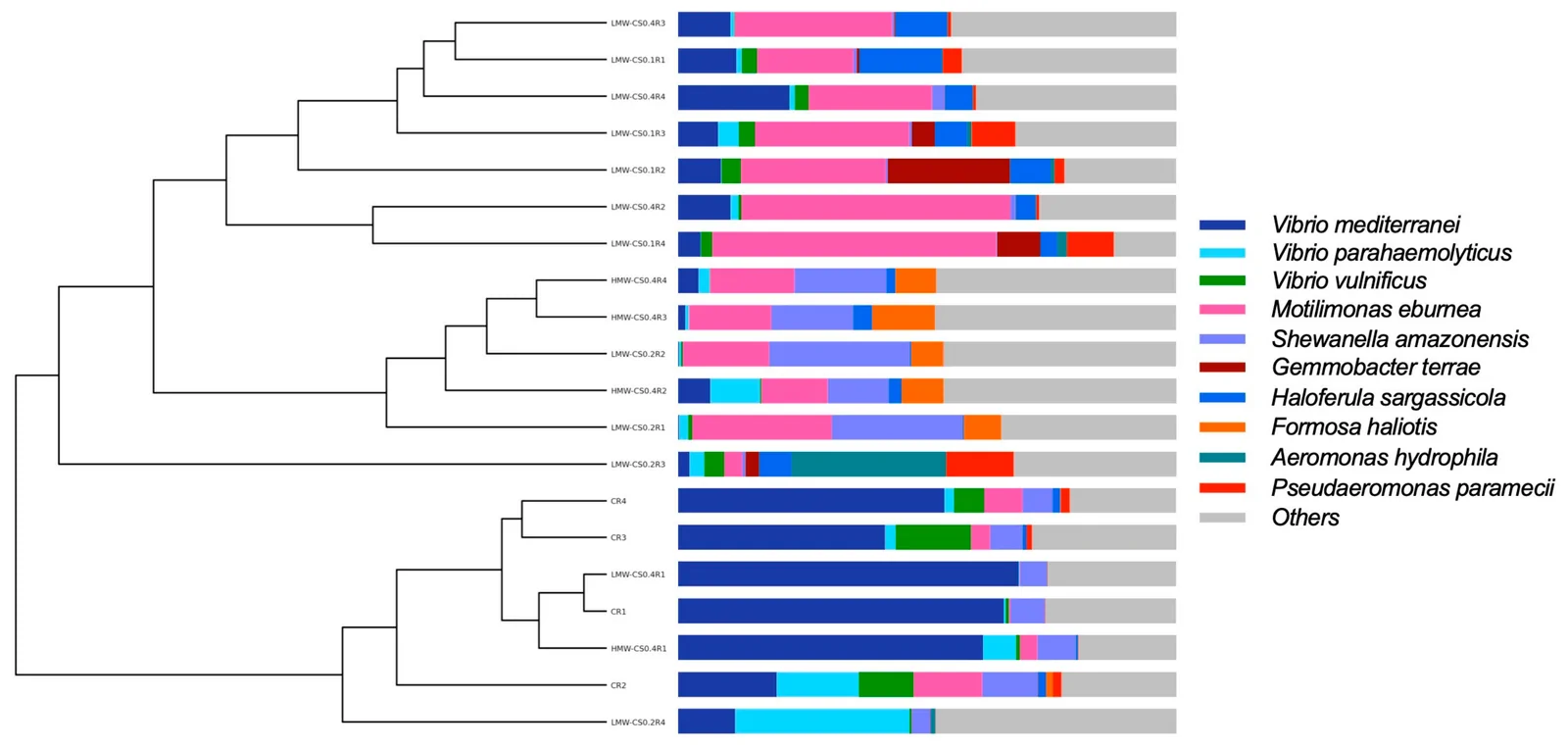
UPGMA clustering combined with species-level taxonomic composition of bacterial communities in Pacific white shrimp (*L. vannamei*) fed control (C), HMW-CS0.4, LMW-CS0.1, LMW-CS0.2, and LMW-CS0.4 diets for 4 weeks. The dendrogram shows similarities in bacterial community composition among individual samples, while stacked bars represent the relative abundance of dominant bacterial species. Four independent intestinal samples were analyzed per treatment (*n* = 4 shrimp, one shrimp sampled from each replicate tank).

UPGMA hierarchical clustering demonstrated treatment-associated differences in bacterial community composition. Most control (C) samples clustered within the lower branch and were separated from the majority of chitosan-treated samples. Among the chitosan groups, LMW-CS0.1 exhibited the most consistent clustering pattern, with all replicates grouped within the upper branch. Most LMW-CS0.4 samples were also located in this branch, indicating a close compositional similarity between LMW-CS0.1 and LMW-CS0.4. By contrast, HMW-CS0.4 and LMW-CS0.2 showed greater replicate-level dispersion, with some samples positioned near the C cluster. Overall, UPGMA clustering showed that LMW-CS supplementation, particularly at 0.1% and 0.4% *w*/*w*, was associated with a distinct shift in bacterial community structure relative to the control. These clustering patterns are descriptive and should not be interpreted as statistically confirmed differences among dietary treatments (Figure 8).

#### 3.7.3. *16S* Functional Genes Prediction

**Figure 9:**
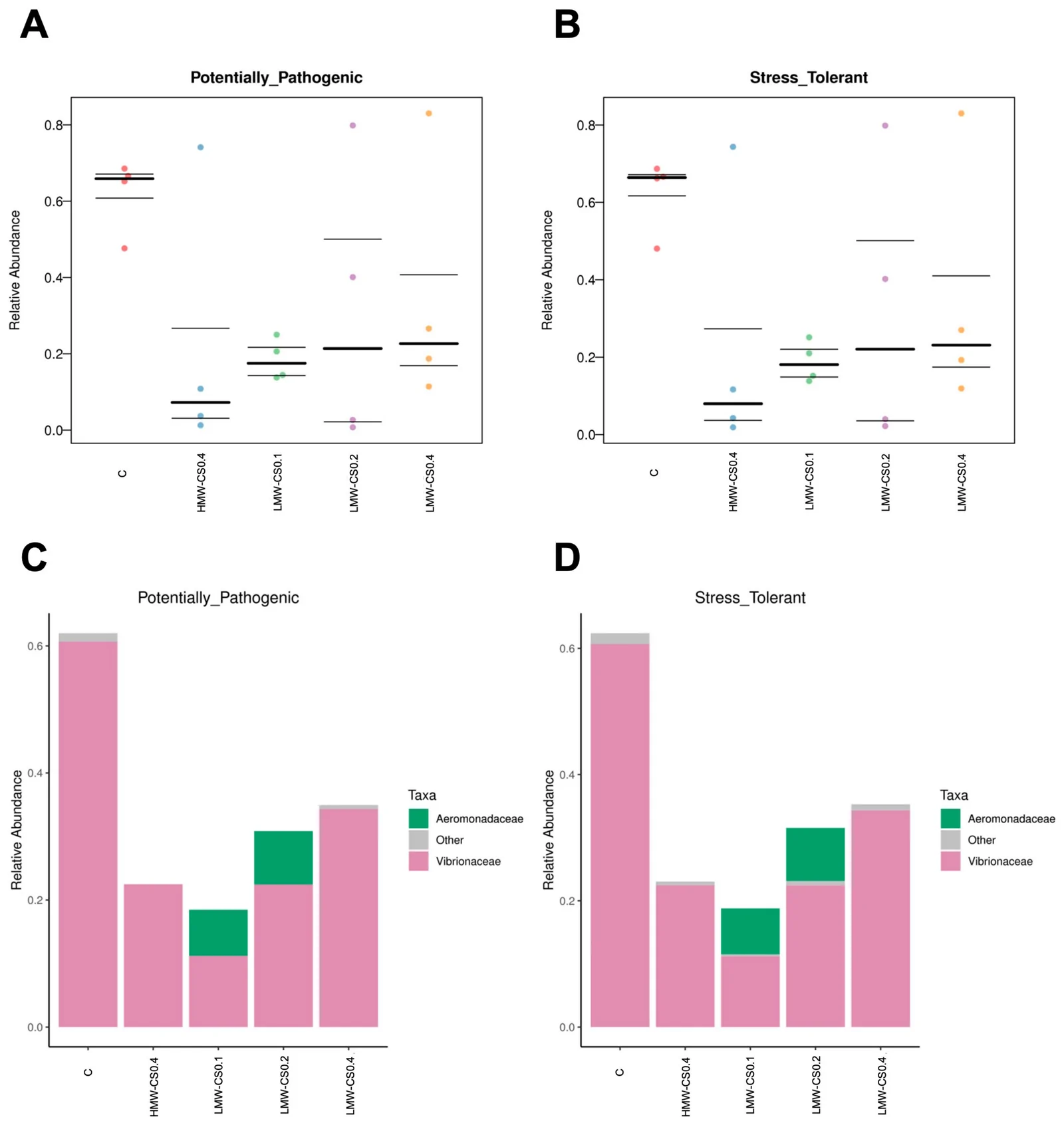
Phenotypic prediction of bacterial communities in Pacific white shrimp (*L. vannamei*) fed control and chitosan-supplemented diets for 4 weeks based on BugBase analysis. Relative abundance of bacterial communities predicted as potentially pathogenic (**A**) and stress-tolerant (**B**) among treatment groups. Relative abundance of family-level taxa contributing to potentially pathogenic (**C**) and stress-tolerant (**D**) phenotypes. C: basal diet; HMW-CS0.4: basal diet supplemented with non-degraded high-molecular-weight chitosan at 0.4% *w*/*w*; LMW-CS0.1, LMW-CS0.2, and LMW-CS0.4: basal diet supplemented with degraded low-molecular-weight chitosan at 0.1%, 0.2%, and 0.4% *w*/*w*, respectively. Four independent intestinal samples were analyzed per treatment (*n* = 4 shrimp, one shrimp sampled from each replicate tank).

BugBase analysis revealed treatment-associated differences in predicted bacterial phenotypes. The control group showed the highest predicted proportions of both potentially pathogenic and stress-tolerant phenotypes, whereas all chitosan-supplemented groups showed lower mean values. Among the treatments, LMW-CS0.1 consistently exhibited the lowest predicted abundance for both phenotypes, followed by HMW-CS0.4, while LMW-CS0.2 and LMW-CS0.4 showed intermediate levels. However, Kruskal–Wallis tests indicated no significant overall differences among groups, and pairwise differences were not significant after FDR correction. Therefore, these results indicate a descriptive reduction rather than a statistically confirmed effect (Figure 9A,B).

At the family level, Vibrionaceae was the major contributor to both predicted phenotypes, especially in the control group. Chitosan supplementation reduced the relative contribution of Vibrionaceae-associated phenotypes, particularly in HMW-CS0.4 and LMW-CS0.1. Minor contributions from Aeromonadaceae and other taxa were also observed, with relatively greater non-Vibrionaceae contributions in some LMW-CS groups. Collectively, these findings suggest that dietary chitosan, especially LMW-CS0.1, may shift bacterial communities toward lower predicted pathogenic and stress-tolerant potential, mainly through reduced Vibrionaceae-associated signals. (Figure 9C,D).

#### 3.7.4. Bacterial Load and Survival After *V. parahaemolyticus* Challenge

(1) Bacterial load

**Figure 10:**
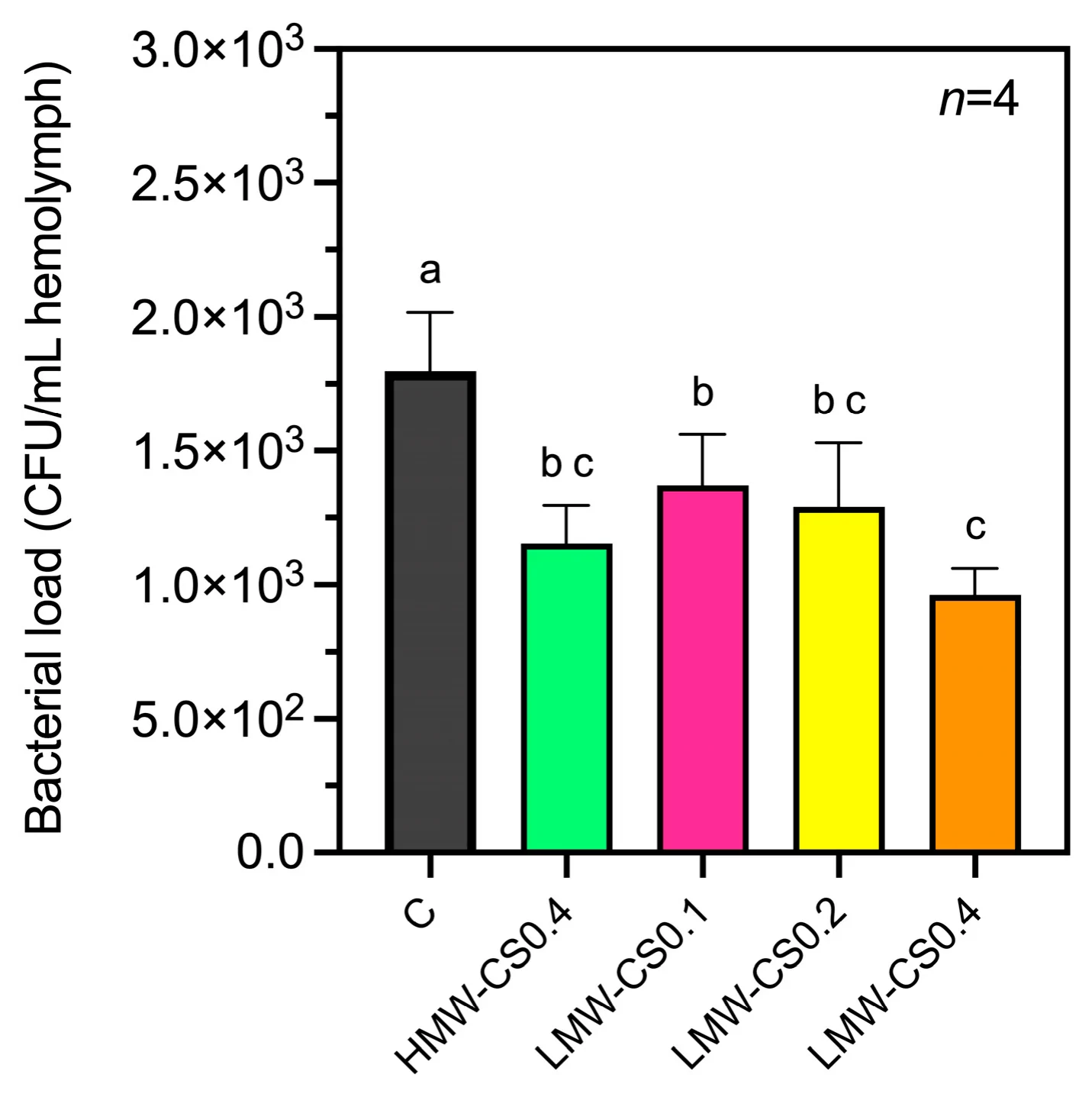
Effects of dietary chitosan on bacterial load in Pacific white shrimp (*L. vannamei*) after *Vibrio parahaemolyticus* challenge in shrimp fed control, HMW-CS0.4, LMW-CS0.1, LMW-CS0.2, and LMW-CS0.4 diets for 4 weeks. Data are presented as mean ± SD of four independent biological replicates per treatment (*n* = 4 shrimp, one shrimp sampled from each replicate challenge tank). Different lowercase letters indicate significant differences among treatments (*p* < 0.05).

Bacterial load after *Vibrio parahaemolyticus* challenge was significantly affected by dietary treatment (*p* = 0.0002). The control group showed the highest bacterial load at 1.80 ± 0.22 × 10^3^ CFU/mL, whereas the LMW-CS0.4 group showed the lowest bacterial load at 0.96 ± 0.10 × 10^3^ CFU/mL. Shrimp fed HMW-CS0.4, LMW-CS0.1, and LMW-CS0.2 also showed significantly lower bacterial loads than the control group, with mean values of 1.16 ± 0.14, 1.37 ± 0.19, and 1.29 ± 0.24 × 10^3^ CFU/mL, respectively (Figure 10).

(2) Survival after *Vibrio parahaemolyticus* challenge

**Figure 11:**
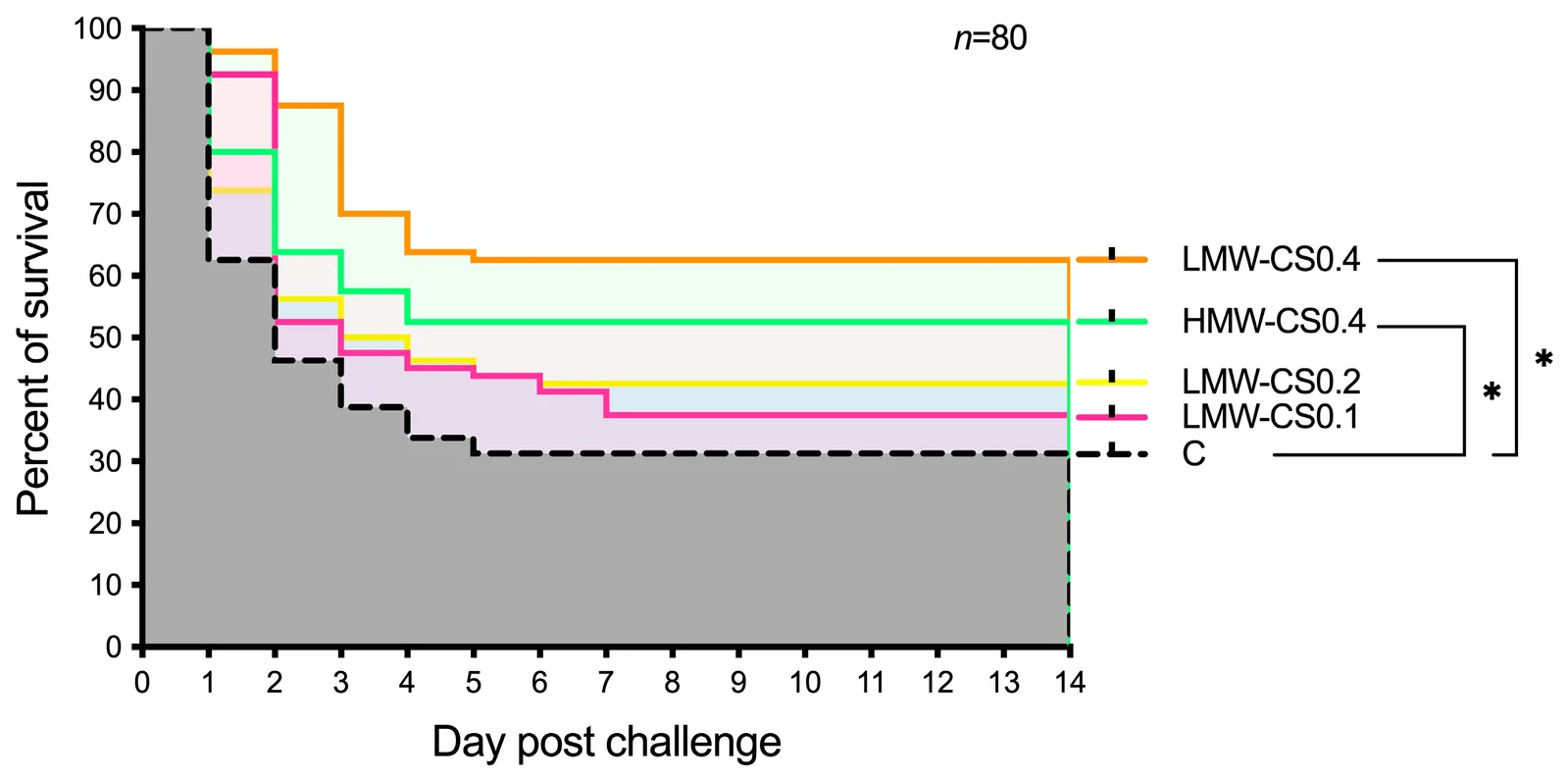
Survival of Pacific white shrimp (*L. vannamei*) after *Vibrio parahaemolyticus* challenge. Shrimp were fed control, HMW-CS0.4, LMW-CS0.1, LMW-CS0.2, or LMW-CS0.4 diets for 4 weeks before bacterial challenge. Survival was monitored for 14 days post-challenge. Asterisks indicate significant differences among treatments (*p* < 0.05). Each treatment included four replicate tanks with 20 shrimp per tank, giving 80 shrimp per treatment (*n* = 80 shrimp).

Dietary chitosan supplementation improved shrimp survival after *Vibrio parahaemolyticus* challenge. Survival curves differed significantly among treatments based on the log-rank Mantel–Cox test (*p* < 0.0001). The highest final survival was observed in the LMW-CS0.4 group at 62.5%, followed by HMW-CS0.4 at 52.5%, LMW-CS0.2 at 42.5%, LMW-CS0.1 at 37.5%, and the control group at 31.3%. Median survival was shortest in the control group at 2 days, while HMW-CS0.4 and LMW-CS0.4 showed extended median survival to 14 days. These results indicate that dietary LMW-CS, particularly at 0.4%, enhanced the resistance of Pacific white shrimp against *V. parahaemolyticus* infection (Figure 11).

## 4. Discussion

The production of LMW-CS through γ-irradiation in the presence of hydrogen peroxide may have contributed to its enhanced biological performance. Gamma irradiation induces chain scission of chitosan, while H_2_O_2_ enhances radical formation and accelerates depolymerization. Hien et al. [34] demonstrated that γ-irradiation in the presence of H_2_O_2_ efficiently degraded chitosan in solution and reduced its molecular weight, supporting the synergistic degradation approach used in the present study. The resulting lower-molecular-weight chitosan may have improved solubility and bioavailability, allowing more effective interaction with shrimp tissues and immune-related pathways.

The present study demonstrated that dietary supplementation with synergistically degraded low-molecular-weight chitosan improved growth performance, antioxidant status, innate immune responses, immune-related gene expression, bacterial clearance, and survival of Pacific white shrimp (*L. vannamei*) after *Vibrio parahaemolyticus* challenge. These findings indicate that the biological efficacy of chitosan was strongly influenced by molecular weight and inclusion level. The improved responses observed in shrimp fed LMW-CS0.2 and LMW-CS0.4 suggest that degraded low-molecular-weight chitosan had greater functional activity than native high-molecular-weight chitosan. This agrees with previous evidence that the bioactivity of chitooligosaccharides in *L. vannamei* is affected by dosage, molecular weight, and degree of deacetylation, with optimized forms improving growth performance, innate immunity, antioxidant status, and hepatopancreas morphology [35].

The improved growth performance and feed utilization observed in the LMW-CS0.2 and LMW-CS0.4 groups may be related to the physicochemical properties of degraded chitosan. Low-molecular-weight chitosan generally has lower viscosity, better solubility, improved dispersion, and more accessible functional groups than high-molecular-weight chitosan. These characteristics may enhance its interaction with the intestinal surface, improve nutrient absorption, and promote physiological conditions. However, these potential mechanisms were not directly evaluated in the present study and should be confirmed through intestinal histological analysis and nutrient-transporter expression assays. Similar growth-promoting effects were reported by Liu et al. [35], who found that chitooligosaccharide supplementation improved growth performance and feed utilization in Pacific white shrimp, depending on molecular weight and dietary dose. Therefore, the improved final weight, total weight gain, ADG, and FCR in the present study may reflect enhanced feed efficiency and better metabolic status rather than only a direct growth-stimulating effect.

Dietary chitosan also enhanced innate humoral immunity, as shown by increased lysozyme activity. Lysozyme is an important nonspecific immune enzyme involved in bacterial cell wall degradation and pathogen clearance. In the present study, lysozyme activity was highest in the LMW-CS0.4 and HMW-CS0.4 groups, indicating that chitosan supplementation at sufficient inclusion levels stimulated humoral antibacterial defense. This result agrees with Liang et al. (2020), who reported that oral administration of chitosan-gentamicin conjugate enhanced nonspecific immune parameters, including lysozyme activity, in *L. vannamei* after *V. parahaemolyticus* infection [36]. However, unlike that study, the present work used degraded chitosan alone without antibiotic conjugation, suggesting that LMW-CS itself can act as an immunonutritional additive.

The gene expression results further support the immunostimulatory role of LMW-CS. The upregulation of *igf2*, particularly in the LMW-CS0.4 group, may be associated with improved growth and metabolic regulation. The induction of *cstn* and *lyz* indicates activation of antimicrobial defense, while the strong upregulation of *lgbp* suggests enhanced pathogen recognition. In crustaceans, lipopolysaccharide- and β-1,3-glucan-binding protein acts as a pattern-recognition molecule that detects microbial components and initiates downstream immune responses. Maralit et al. [37] reported that hemocytes of *L. vannamei* challenged with AHPND-causing *V. parahaemolyticus* showed differential expression of immune-related genes, including transcripts associated with antimicrobial defense and pathogen recognition. Therefore, the increased expression of *cstn*, *lgbp*, and *lyz* in the present study suggests that LMW-CS primed shrimp immune readiness against bacterial infection.

The prophenoloxidase system is a key innate immune pathway in shrimp, contributing to melanization, pathogen encapsulation, and microbial killing. In the present study, hepatopancreatic *propo2* expression was strongly upregulated in all chitosan-supplemented groups, whereas *propo1* showed only minor changes. This indicates that *propo2* may be more responsive to dietary chitosan stimulation under the present experimental conditions. The activation of prophenoloxidase-related responses may have contributed to the reduced bacterial load and improved survival after the *V. parahaemolyticus* challenge. This interpretation is supported by Liang et al. [38], who demonstrated that chitosan-gentamicin conjugate improved the resistance of Pacific white shrimp to *V. parahaemolyticus* infection through immune regulation.

The antioxidant results showed that LMW-CS supplementation reduced MDA levels and increased GSH, NO, GR, CAT, SOD, GPx, and GST activities in both plasma and hepatopancreas. MDA is a major indicator of lipid peroxidation, and its reduction indicates lower oxidative damage. In contrast, increased GSH and antioxidant enzyme activities suggest enhanced redox buffering and detoxification capacity. These results are consistent with Liu et al. [35], who reported that chitooligosaccharide supplementation improved antioxidant capacity and hepatopancreatic condition in *L. vannamei*. The hepatopancreas is highly sensitive to oxidative stress because it is involved in digestion, metabolism, detoxification, and immune regulation. Therefore, the reduction in MDA together with the enhancement of antioxidant enzymes suggests that LMW-CS helped protect hepatopancreatic tissues against oxidative injury.

The coordinated increase in antioxidant parameters suggests that LMW-CS enhanced multiple components of the antioxidant defense system. SOD converts superoxide radicals into hydrogen peroxide, while CAT and GPx further detoxify hydrogen peroxide and lipid hydroperoxides. GR maintains the reduced glutathione pool, and GST contributes to detoxification through conjugation reactions. This integrated antioxidant response may explain why shrimp fed LMW-CS0.2 and LMW-CS0.4 showed better physiological performance and improved resistance to bacterial challenge. Similar antioxidant enhancement has been reported in shrimp treated with chitosan-based materials, including chitosan-gentamicin conjugates, which increased antioxidant-related immune parameters in *L. vannamei* infected with *V. parahaemolyticus* [36].

Dietary chitosan supplementation modified the intestinal bacterial community of Pacific white shrimp mainly through changes in community composition rather than overall diversity. Although alpha diversity indices remained unchanged, PCoA and UPGMA analyses showed separation between the control and chitosan-treated groups, indicating selective reshaping of the bacterial community. Proteobacteria remained the dominant phylum across treatments, consistent with previous reports that this phylum is a major component of the *L. vannamei* gut microbiota [39]. The most evident taxonomic change was the reduction in *Vibrio* spp., particularly *Vibrio mediterranei* and *V. vulnificus*, in shrimp fed LMW-CS. This is relevant because *Vibrio* is commonly detected in shrimp gut communities, but several members of this genus are opportunistic pathogens associated with shrimp disease and microbiota dysbiosis [40,41,42]. The distinct microbial profile observed in the LMW-CS groups is consistent with previous studies reporting stronger antimicrobial activity of low-molecular-weight chitosan than its high-molecular-weight counterpart [43].

The BugBase results were consistent with these taxonomic shifts. The lower predicted abundance of potentially pathogenic and stress-tolerant phenotypes in chitosan-treated groups, particularly LMW-CS0.1, was mainly linked to the reduced contribution of Vibrionaceae. Although these phenotype predictions were not statistically significant, they followed the same trend as the species-level data, suggesting that the decline in *Vibrio*-associated taxa was reflected in the predicted functional profiles. As BugBase relies on 16S rRNA gene-based inference, these results should be interpreted as supportive rather than direct functional evidence.

The improved disease resistance observed after the *V. parahaemolyticus* challenge provides functional confirmation of the antioxidant and immune results. Shrimp fed LMW-CS0.4 showed the highest survival and the lowest bacterial load, while LMW-CS0.2 also showed strong protective effects. This indicates that LMW-CS enhanced host defense through multiple mechanisms, including reduced oxidative damage, enhanced antioxidant enzyme activity, increased lysozyme activity, upregulation of antimicrobial genes, stimulation of pathogen recognition, and activation of prophenoloxidase-related pathways. These findings are consistent with published studies showing that chitosan-based products can improve the resistance of *L. vannamei* to *V. parahaemolyticus*. For example, Liang et al. [36] reported that chitosan-gentamicin conjugate improved nonspecific immunity, intestinal condition, and microbial balance in infected shrimp, while Liang et al. [38] further described its therapeutic and immune-regulatory effects during *V. parahaemolyticus* infection.

Compared with previous studies, the present study is distinct because the functional additive was produced by synergistic γ-irradiation and H_2_O_2_ degradation and was applied without antibiotic conjugation or additional encapsulated compounds. Previous work has shown that chitooligosaccharides can improve growth, immunity, antioxidant capacity, and hepatopancreatic morphology in *L. vannamei* (Liu et al. [35]), and chitosan-antibiotic conjugates can improve resistance to *V. parahaemolyticus* infection [36,38]. However, the present findings show that synergistically degraded LMW-CS alone can promote growth, antioxidant defense, innate immunity, and disease resistance. This supports its potential as an antibiotic-free functional feed additive for sustainable shrimp aquaculture.

The dose-dependent pattern observed in this study is also important. LMW-CS0.2 was highly effective in improving growth performance, antioxidant status, and hepatopancreatic *lyz* expression, while LMW-CS0.4 showed stronger effects on lysozyme activity, immune gene activation, bacterial load reduction, and survival. In contrast, LMW-CS0.1 produced weaker and less consistent effects, suggesting that this level may be insufficient to fully activate antioxidant and immune pathways. These findings indicate that the optimal inclusion level of LMW-CS may depend on the production objective. LMW-CS0.2 may be suitable for growth and antioxidant enhancement, whereas LMW-CS0.4 may be more effective for strengthening immune defense and resistance to bacterial infection.

Overall, the present study indicates that synergistically degraded LMW-CS is a promising functional feed additive for Pacific white shrimp. Its beneficial effects are likely mediated by improved physicochemical availability, enhanced redox defense, stimulation of antimicrobial and pathogen-recognition pathways, activation of the prophenoloxidase system, improved bacterial clearance, and increased survival after *V. parahaemolyticus* challenge. These results support the use of LMW-CS as a sustainable, antibiotic-free strategy to improve shrimp health and resilience against AHPND-associated *V. parahaemolyticus* in intensive aquaculture systems.

A limitation of the present study is that biochemical, immune, gene-expression, bacterial-load, and microbiota measurements were based on four independent biological replicates per treatment, with one shrimp sampled from each replicate tank. Although this design preserved independence among biological replicates and avoided pseudoreplication, sampling additional shrimp within each tank would have provided more precise estimates of within-tank variability and improved power to detect smaller treatment effects. Future studies should include multiple shrimp per tank and an a priori power analysis while retaining the tank as the experimental unit or accounting for tank-level clustering in the statistical model.

## 5. Conclusions

This study demonstrated that synergistically degraded low-molecular-weight chitosan (LMW-CS) is an effective functional feed additive for Pacific white shrimp (*L. vannamei*). Dietary LMW-CS improved growth performance, feed utilization, antioxidant defense, lysozyme activity, immune-related gene expression, gut microbial balance, and resistance to *Vibrio parahaemolyticus*. LMW-CS0.2 (0.2% *w*/*w*) was most effective for promoting growth and feed efficiency, whereas LMW-CS0.4 (0.4% *w*/*w*) provided the strongest protection after bacterial challenge, as shown by reduced bacterial load and improved survival. However, this study was conducted under controlled laboratory conditions over a four-week feeding period, and intestinal morphology, nutrient absorption, and microbial functions were not directly evaluated. Therefore, further long-term and farm-scale studies are required to confirm the efficacy and underlying mechanisms of dietary LMW-CS. These findings indicate that molecular-weight reduction enhances the biological functionality of chitosan and support the use of dietary LMW-CS as an antibiotic-free strategy to improve shrimp health and disease resilience in sustainable aquaculture.

## Figures and Tables

**Table 1:** Primers used for gene expression analysis in this study.

Gene Group	Genes	Primer Names	Nucleotide Sequences (5′→3′)	Tm (°C)	Accession Number/Reference
Growth-related gene	*Insulin-like growth factor 2* (*igf2*)	*Lv_igf2*	F: CTCTGTACAGTCAGCCCAGC R: CACACCCAGTCAGTCCCAAG	60	[26]
Antimicrobial peptide/ immune-related gene	*Crustin-like antimicrobial peptide* (*cstn*)	*Lv_cstn*	F: CACGAGGCAACCATGAAGG R: TCTTGCACCAATACCTGCAGT	60	AF430076
	*Alkaline phosphatase* (*alp*)	*Lv_alp*	F: GGCGGTCAGAGTGGAGAT R: CGCAATGCTGTAGAAGGAC	60	KR534873
	*Lysozyme* (*lyz*)	*Lv_lyz*	F: GGACTACGGCATCTTCCAGA R: ATCGGACATCAGATCGGAAC	55	AY170126
Pattern-recognition receptor gene	*Lipopolysaccharide- and β-1,3-glucan-binding protein* (*lgbp*)	*Lv_lgbp*	F: ACCGCAGCATCAGTTATACC R: GTCATCGCCCTTCCAGTTG	60	AY723297
Prophenoloxidase system-related gene	*Prophenoloxidase 1* (*propo1*)	*Lv_propo1*	F: CCTCACAGGCTGGAACACAA R: GGCGAAGAATCACGGGTCTA	60	EF115296
	*Prophenoloxidase 2* (*propo2*)	*Lv_propo2*	F: GTTGGAGGCCGACTCGAAT R: AATGAGGACGTGACCCATGTT	60	EF565469
Immune signaling/ cellular response-related gene	*Ras-related protein Rap-2a* (*rap2a*)	*Lv_rap2a*	F: GCCGTGCGTGCTTGAGAT R: TTGATGTCCTGGAAGGTCTGG	55	[27]
Antioxidant-related gene	*Superoxide dismutase* (*sod*)	*Lv_sod*	F: TCATGCTTTGCCACCTCTC R: CCGCTTCAACCAACTTCTTC	55	AY486424
Reference genes	*Beta-actin* (*actb*)	*Lv_actb*	F: TCCACGAGACCACCTACAACTC R: GAGGGCAGTGATTTCCTTCTG	56	AF300705
	*Elongation factor 1-alpha* (*ef1a*)	*Lv_ef1a*	F: TGGCTACTCACCTGTGCTTG R: CCAGCTCCTTACCAGTACGC	60	GU136229
	*Glyceraldehyde-3-phosphate dehydrogenase* (*gapdh*)	*Lv_gapdh*	F: CCATGGAATGTTCTCGGGCT R: AAGTATGACAGCACCCACGG	60	KT861451

## Data Availability

The original contributions presented in this study are included in the article. Further inquiries can be directed to the corresponding author.
